# Biologically Targeted Photo‐Crosslinkable Nanopatch to Prevent Postsurgical Peritoneal Adhesion

**DOI:** 10.1002/advs.201900809

**Published:** 2019-08-13

**Authors:** Yu Mi, Feifei Yang, Cameron Bloomquist, Youli Xia, Bo Sun, Yanfei Qi, Kyle Wagner, Stephanie A. Montgomery, Tian Zhang, Andrew Z. Wang

**Affiliations:** ^1^ Laboratory of Nano‐ and Translational Medicine Carolina Center for Cancer Nanotechnology Excellence Carolina Institute of Nanomedicine Lineberger Comprehensive Cancer Center Department of Radiation Oncology University of North Carolina at Chapel Hill Chapel Hill NC 27599 USA; ^2^ Institute of Medicinal Plant Development (IMPLAD) Chinese Academy of Medical Sciences & Peking Union Medical College Haidian District Beijing 100193 P. R. China; ^3^ School of Pharmacy University of North Carolina at Chapel Hill Chapel Hill NC 27599 USA; ^4^ Department of Genetics University of North Carolina at Chapel Hill Chapel Hill NC 27599 USA; ^5^ School of Public Health Jilin University Changchun Jilin 130021 P. R. China; ^6^ Department of Pathology and Laboratory Medicine University of North Carolina at Chapel Hill Chapel Hill NC 27599 USA; ^7^ Department of Medical Oncology Department of Medicine Duke University Medical Center Durham NC 27710 USA

**Keywords:** abdominal surgery, biological targeting, nanomaterials, peritoneal adhesion, photo‐crosslinking

## Abstract

Peritoneal adhesion occurs in a majority of patients following abdominal surgery and can result in significant side effects and complications. Current strategies to minimize adhesions involve the use of nontargeted anatomical barriers that are either inefficient in protecting injured areas or lacking the adequate residence time to prevent adhesions. Herein, the development of a biologically targeted photo‐crosslinkable nanopatch (pCNP) is reported that can prevent postsurgical adhesion. It is demonstrated that pCNP can form a compact protective barrier over surfaces with exposed collagen IV. Using a rat parietal peritoneal excision adhesion model, it is showed that pCNP is highly effective and safe in preventing postsurgical adhesions. This work presents a novel approach to preventing peritoneal adhesion with nanomaterials.

Abdominal surgery is an important treatment in many diseases, such as cancers and inflammatory bowel diseases. A frequent side effect of abdominal surgery is the formation of peritoneal adhesions.^1^, ^2^ It has been reported that up to 93% of patients who underwent abdominal surgery were found to have postsurgical adhesion.^3^, ^4^, ^5^ Such adhesions can cause pain, bowel obstruction as well as other serious complications.^6^ The biology of adhesion formation is highly complex, involving many chemical mediators, cytokines, and cell types.^7^ It is thought to be due to the imbalance between inflammatory and healing processes.^8^, ^9^ Pro‐inflammatory processes, such as macrophage activation and fibroblast activation, are known to play an important role in adhesion formation.^10^, ^11^

Current strategies to minimize adhesions mainly involve the use of anatomical barriers between injured peritoneal surfaces to prevent adhesion formation. Such barriers include adhesion‐reducing liquids and polymer‐based barriers (e.g., cellulose) that are in the form of gels or films.^7^, ^12^, ^13^, ^14^ Adhesion‐preventing liquids generally contain polymers such as icodextrin or polyethylene glycol (PEG). They function by occupying the abdominal cavity and allowing injured surfaces to heal undisturbed. A recent meta‐analysis showed that the use of adhesion barriers (both liquids and films/gels) in abdominal surgery likely reduces the formation of adhesions.^15^ However, the effects are moderate. Moreover, for several formulations, including icodextrin, PEG, and oxidized regenerated cellulose, there is no clear evidence that they can reduce the complications resulting from postoperative adhesions. The lack of efficacy from adhesion‐preventing liquids is likely due to their absorption by the peritoneal cavity, thus not providing a persistent barrier on injured surfaces. Polymer films and gels have been used as barriers but these nontargeted and bulk barriers are not always able to cover all injured peritoneal surfaces. Moreover, these barriers do not always stay in place postsurgery, thus limiting their therapeutic efficacy. Therefore, there has been strong interest in the development of strategies and agents that can prevent postsurgical adhesion formation.

Advances in nanotechnology have enabled the development of nanomaterials that can be biologically targeted toward specific diseases processes.^16^, ^17^, ^18^, ^19^ Here we report the development of a biologically targeted, photo‐crosslinkable nanopatch (pCNP) for postsurgical adhesion prevention. pCNP is comprised of two NPs. The first NP (NP‐A) is designed to carry the anti‐inflammatory cargo^20^, ^21^, ^22^ and specifically bind to the injury site. Since the hallmark of injured epithelial/mesothelial surfaces is exposed basement membranes, and a key component of basement membrane is collagen IV, we chose to target NP‐A against collagen IV. The second NP (NP‐B) is designed with a positively charged surface, opposite to that of NP‐A, to enable absorption to the layer of NP‐A through ionic interactions (**Figure** **1**a). The two NPs can be subsequently crosslinked by UV‐irradiation through diazirine reactive group to form a nanopatch. The pCNP was designed to be administered intraperitoneally in sequence (Figure 1b). First, a suspension of NP‐A is incubated at the site of injury to allow for specific binding to the basement membrane, which is exposed following mesothelial damage. Particle properties that afford a stable suspension for administration would result in an insufficiently low‐density barrier when a single particle is administered due to steric and ionic hindrance. Thus, a suspension containing NP‐B is subsequently administered to rapidly form a dense layer through ionic adsorption between the oppositely charged NP‐A and NP‐B. The injury site is then irradiated with UV light with a wavelength of 365 nm to initiate crosslinking of the two nanoparticles, forming a specific and dense biological barrier between the injured peritoneal surfaces.

**Figure 1:**
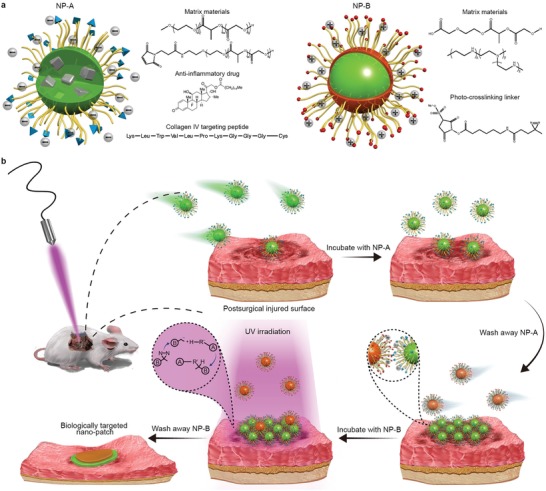
Schematic depiction of photo‐crosslinkable nanopatch (pCNP). a) Designs of the two nanoparticles that comprise pCNP. Green: PLGA polymer; Yellow: PEG polymer; Gray: anti‐inflammatory drug; Blue: collagen IV‐targeting peptide; Brown: PEI polymer; Red: photo‐crosslinkable group. b) schematic showing the formation of pCNP on an injured surface to prevent postsurgical peritoneal adhesion.

NP‐A was formulated using a poly(ethylene glycol)‐poly(lactic‐*co*‐glycolic acid) block copolymer (PEG‐PLGA), which was functionalized with a collagen IV‐targeting peptide. The collagen IV‐targeting peptide functionalized PEG‐PLGA (Col‐PEG‐PLGA) was characterized by 1H NMR (Figure S1, Supporting Information). Dexamethasone 21‐Palmitate (Dex‐Pal), an anti‐inflammatory agent to prevent adhesion formation,^17^, ^23^, ^24^ was encapsulated into NP‐A with the loading amount of 65.2 ± 4.02 µg mg^−1^. Physical characterization of NP‐A demonstrated a spherical morphology with an average hydrodynamic diameter of 166.1 ± 1.8 nm and a negatively charged surface of −12.1 ± 0.3 mV. NP‐B was formulated to contain a PLGA‐PEG core and a branched polyethyleneimine (PEI) shell. The surface of NP‐B was functionalized to display diazirine groups to allow photo‐induced crosslinking among NPs. NP‐B was characterized with an average hydrodynamic diameter of 175.1 ± 28.7 nm and a positively charged surface of 23.0 ± 3.0 mV.

To confirm the ability of pCNP to form a nanopatch, we first verified the crosslinking reaction between NP‐A and NP‐B using photo‐DSC analysis (Figure S2, Supporting Information). We found that the peak of reaction emerged around 1.5 minutes after the exposure of UV irradiation and the reaction was complete within 10 minutes. As expected, the heat of the reaction appeared to be dependent on the concentration of NP‐B, and thus the diazirine groups. Notably, no detectable heat was produced in water without NP‐A or NP‐B by UV irradiation.

We then demonstrated that the NP regimen can form a dense and stable nanopatch in a biologically targeted fashion in vitro. NPs were applied to noncoated or collagen‐IV‐coated glass slides and the formation of the nanopatch was examined. A collagen IV surface was used to simulate injured peritoneal surfaces, as collagen IV is one of the major constituents of basement membrane. The density of the nanopatch was confirmed by field emission scanning electron microscope (FESEM) (**Figure** **2**a–p, Figure S3, Supporting Information) and quantified using the integrated gray value of the FESEM images (Figure 2q,r). We found that nanopatch density formed by biologically targeted NPs (NP‐A) was ≈2.1 times (average times of integrated density of gray value in 10k and 20k magnified FESEM images) higher than that of nontargeted (NP‐A′) on collagen‐IV‐coated surface. Additionally, the density of pCNP on collagen‐IV‐coated surface was ≈2.6 times higher than that of NP‐A only and ≈3.1 times higher than NP‐B only, indicating that pCNP was more efficient in forming high nanopatch density than either of its constituent NPs. The density of pCNP on collagen‐IV‐coated surface was ≈3.0 times higher than that on noncoated surface, showing the specificity of pCNP to collagen IV‐enriched surface. Here, both NP‐A and NP‐B bind with the collagen‐IV‐coated surface. The layer of NP‐A is formed by specific binding between NP‐A and collagen IV. The layer of NP‐B is formed by photo‐crosslinking between NP‐B and collagen IV. However, either NP‐A or NP‐B suffers low density due to steric and ionic hindrance, which is consistent with our hypothesis. Therefore, both NP‐A and NP‐B are required to form an effective nanopatch barrier. We checked the retention and degradation of pCNP on collagen IV‐coated glass cover slides in PBS at 37 °C. It showed that pCNP can last as a dense patch on collagen‐IV‐coated surface for two weeks with a decrease in density of 25% (Figure S4, Supporting Information). Our findings demonstrated the importance of the targeting ligand, the presence of NP‐B, and UV‐induced crosslinking in achieving a high and durable nanopatch density.

**Figure 2:**
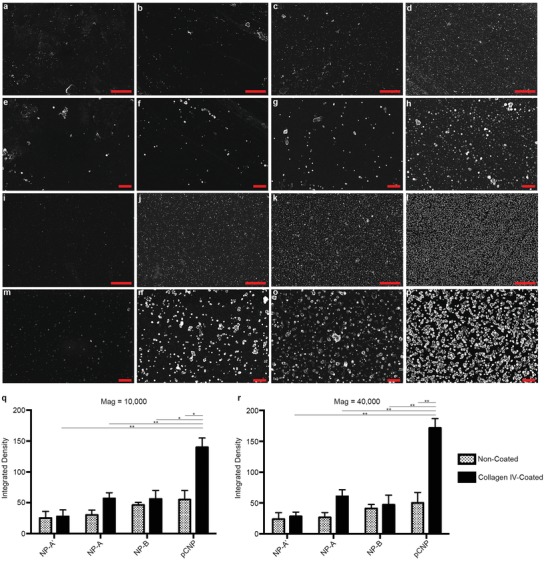
pCNP forms a high‐density nanopatch on collagen IV‐enriched surface in vitro. a–p) FESEM images showing the formation of a nanopatch on noncoated (a–h) and collagen‐IV‐coated (i–p) glass cover slides using different approaches. a,e,i,m) NP‐A without targeting ligand (NP‐A′); b,f,j,n) NP‐A; c,g,k,o) NP‐B; d,h,l,p) pCNP. a–d) and i–l) FESEM images at 10 000 magnification. Scale bar = 5 µm. e–h) and m–p) FESEM images at 40 000 magnification. Scale bar = 1 µm. q) Integrated density of gray value in (a–d), (i–l), and Figure S2 in the Supporting Information. Data represents mean ± standard error of the mean (SEM) (*n* = 3). *P < 0.05, **P < 0.01. r) Integrated density of gray value in (e–h), (m–p), and Figure S2 in the Supporting Information. Data represents mean ± standard error of the mean (SEM) (*n* = 3). *P < 0.05, **P < 0.01.

Next, we characterized the release profile and the safety of pCNP in vitro. The pCNP was formed on collagen‐IV‐coated glass cover and incubated in PBS at 37 °C. The release of Dex‐Pal and its active form drug dexamethasone (Dex) was tested with or without the emergence of esterase (Figure S5, Supporting Information). A burst release of 13.5% for Dex‐Pal and 19.1% for Dex was observed within 24 h, which can reduce inflammation. Comparing it to the release profile of NP‐A revealed that crosslinking of NP‐A and NP‐B delayed the release of encapsulated drugs and trapped the released drugs within the nanopatch. The sustained release of Dex‐Pal and Dex over two weeks would further provide anti‐inflammation during the healing process. To confirm the safety of pCNP, we performed MTS assay using NIH/3T3 fibroblast cells on collagen‐IV‐coated 96‐well plate. The cell viability at 2, 24, 72 h after treatment by pCNP with or without UV irradiation was above ≈80% (Figure S6, Supporting Information), indicating a safe usage of pCNP as well as the UV irradiation.

We examined the pCNP's ability to prevent postsurgical peritoneal adhesion in vivo (**Figure** **3**). The rat parietal peritoneum excision (PPE) model was utilized to study postsurgical adhesion (Figure S7, Supporting Information). Survival surgery was carried out to excise a ≈2 cm × 5 cm patch of peritoneum with the underlying muscle layer from the left abdominal wall. We performed immunohistochemistry (IHC) analysis of the abdominal wall in rat PPE model and showed the injured peritoneal surfaces indeed exposes collagen IV, collagen I, and fibronection (Figure S8, Supporting Information). Such finding indicated that our strategy of targeting collagen IV was feasible. Following excision, rats were laid on the side ipsilateral to the excision site in order to expose the injured cavity for NP incubation. A solution of NP‐A was administered to the peritoneal cavity and incubated for 10 minutes. After NP‐A solution was removed, the peritoneal cavity was washed twice with saline. Following NP‐A, NP‐B was administered and incubated for 10 minutes under UV irradiation. The peritoneal cavity was then washed twice with saline. The abdominal surgical wound was then closed with sutures (Figure 3a, Figure S9, Supporting Information). Control experimental groups included saline incubation (PBS group), NP‐A only (A only), NP‐A + NP‐A incubation (A+A), nontargeted NP‐A with NP‐B and UV crosslinking (A′+B), pCNP without encapsulating Dex‐Pal (pCNP w/o Dex), and a commercially available adhesion barrier Seprafilm. To assess the quality and quantity of postsurgical adhesions, a second‐look laparotomy was performed 14 days post surgery. Adhesions were graded based on a previously described scoring system.^25^, ^26^, ^27^

**Figure 3:**
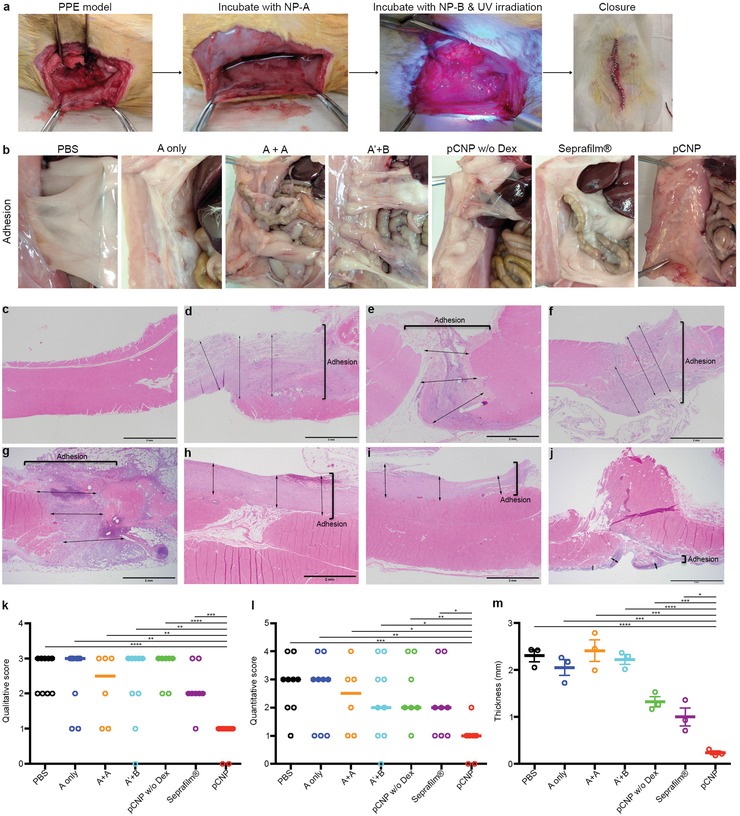
pCNP prevents postsurgical peritoneal adhesion in parietal peritoneal excision (PPE) model in rats. a) Representative photos showing PPE and the administration of pCNP. b) Representative photos demonstrating postsurgical adhesion in rats 14 d after different treatments: PBS, the injured area was incubated with saline for 10 min and another 10 min under UV irradiation; A only, the injured area was incubated with NP‐A for 10 min, washed and incubated with saline for another 10 min under UV irradiation; A+A, the injured area was incubated with NP‐A for 10 min, washed with saline twice, then incubated with NP‐A again for another 10 min under UV irradiation; A′+B, the injured area was incubated with NP‐A′ (NP‐A without targeting ligand) for 10 min, washed with saline twice, then incubated with NP‐B for another 10 min under UV irradiation; pCNP w/o Dex, the injured area was incubated with NP‐A without dexamethasone 21‐palmitate for 10 min, washed with saline twice, then incubated with NP‐B for another 10 min under UV irradiation; Seprafilm, the injured area was incubated with saline for 10 min and another 10 min under UV irradiation, the saline was removed and the injured area was covered with Seprafilm; pCNP, the injured area was incubated with NP‐A for 10 min, washed with saline twice, then incubated with NP‐B for another 10 min under UV irradiation. c–j) Representative H&E staining histology tissue images showing the thickness of adhesion/fibrosis in untreated rats (c), and rats that underwent surgery and subsequent treatment with PBS (d), NP‐A only (e), NP‐A + NP‐A (f), NP‐A′ + NP‐B (g), pCNP without dexamethasone 21‐palmitate (h), Seprafilm (i) and pCNP (j). Scale bar = 2 mm. k,l) Qualitative (k) and quantitative (l) scoring analysis of postsurgical adhesion on rats 14 d after treatments. Statistical significance was assessed using Mann Whitney test. Data represents scatter dot plot with median line (For A+A, *n* = 6; For pCNP w/o Dex, *n* = 7; For Seprafilm, *n* = 8; For other groups, *n* = 9). *P < 0.05, **P < 0.01, ***P < 0.001, ****P < 0.0001. m) Quantitative assessment of the adhesion/fibrosis thickness in (d–j). Statistical significance was assessed using unpaired two‐tailed *t*‐test. Data represents mean ± standard error of the mean (SEM) (*n* = 3). *P < 0.05, **P < 0.01, ***P < 0.001, ****P < 0.0001.

As seen in Figure 3b, the PPE model induces strong postsurgical adhesions in the PBS control group. In contrast, minimal adhesion was seen in rats treated with pCNP (Figure 3b, Figures S10 and S11, Supporting Information). The other experimental groups (A only, A+A, A′+B, pCNP w/o Dex, Seprafilm) demonstrated intermediate levels of adhesion prevention. The levels of adhesions were quantified using a four‐point scale qualitative scoring system (Figure 3k). We demonstrated that pCNP is the most effective treatment in preventing adhesions with a median score of 1, followed by Seprafilm (2), A+A (2.5). Others including PBS, A only, A′+B and pCNP w/o Dex were ineffective in preventing adhesions with scores of 3, and the findings are consistent with the agents' mechanisms of action. We also assessed the quality of adhesions with a five‐point quantitative scoring system (Figure 3l). Similar to the quantity scores, the pCNP treatment was the most effective treatment in preventing adhesions (median score of 1) and PBS was the least effective treatment. All of the NP‐A containing groups were able to reduce the adhesions, likely due to the effects of dexamethasone that was encapsulated within the NPs. Importantly, our observations that pCNP is significantly more effective than NP‐A+NP‐A, NP‐A′+NP‐B, and pCNP w/o Dex demonstrate the importance of crosslinking with NP‐B, biological targeting, and controlled release of Dex‐Pal, respectively. Taken all together, the properties of pCNP made it more efficient in preventing postsurgical peritoneal adhesion than the commercially used adhesion barrier Seprafilm in rat PPE model.

To further quantify the levels of adhesions, we examined the adhesions histologically (Figure 3c–j, Figures S12 and S13, Supporting Information). To compare the adhesions in rats among different treatments, we randomly selected three positions in the histological images and measured the thickness of adhesion/fibrosis. The average thickness of adhesion was 2.31 ± 0.13 mm in PBS group, 2.05 ± 0.17 mm in NP‐A only group, 2.41 ± 0.23 mm in NP‐A plus NP‐A group, 2.23 ± 0.11 mm in NP‐A′ plus NP‐B group, 1.32 ± 0.11 in pCNP w/o Dex group, 1.00 ± 0.19 in Seprafilm group, and 0.24 ± 0.04 mm in pCNP group (Figure 3m). We also explored the Masson's trichrome staining histology tissue images of all the groups, in which the dark blue and thin bands of collagen are natural supporting collagen, meanwhile the disorganized and looser areas of blue are collagen associated with healing process and adhesion/fibrosis (Figure S14, Supporting Information). The result in Masson's trichrome stain indicating the degree of adhesion/fibrosis after different treatments was consistent with that in H&E stain. We demonstrated that pCNP was the most effective treatment in inhibiting postsurgical adhesion with minimal fibrosis in the areas of injury.

Next, we assessed potential systemic adverse events in the rats. Potential side effects include anemia, high WBC from systemic exposure of dexamethasone, bleeding, or infection. Complete blood counts (CBC) were obtained in the animals after PPE surgery. (**Figure** **4**a,b, Figure S15, Supporting Information). The red blood cell count (RBC), hemoglobin (HGB), hematocrit (HCT), and platelets were all within normal ranges after surgery in the experimental arms. An increase of reticulocytes indicated the loss of blood during surgery and RBC recovery after surgery. A decrease of white blood count (WBC) was detected at 24 h, but returned to a normal range after 72 h. A limited decrease of lymphocyte, and increase of neutrophil and monocyte were observed after surgery, consistent with postsurgical reactions. All rats remained alive through the 14 days between the procedure and the second‐look laparotomy with no obvious deterioration of physical symptoms, indicating pCNP was safe in rats. Another potential side effect for Dex‐Pal is increase in blood glucose. We monitored the level of blood glucose in the rats at 6, 24, 48, and 72 h post surgery (Figure 4c). An initial increase was observed for all treatment groups at 6 h after surgery, likely due to stress and glucocorticoid release in response to stress. From then on, the blood glucose decreased and reached normal levels at 72 h after surgery.

**Figure 4:**
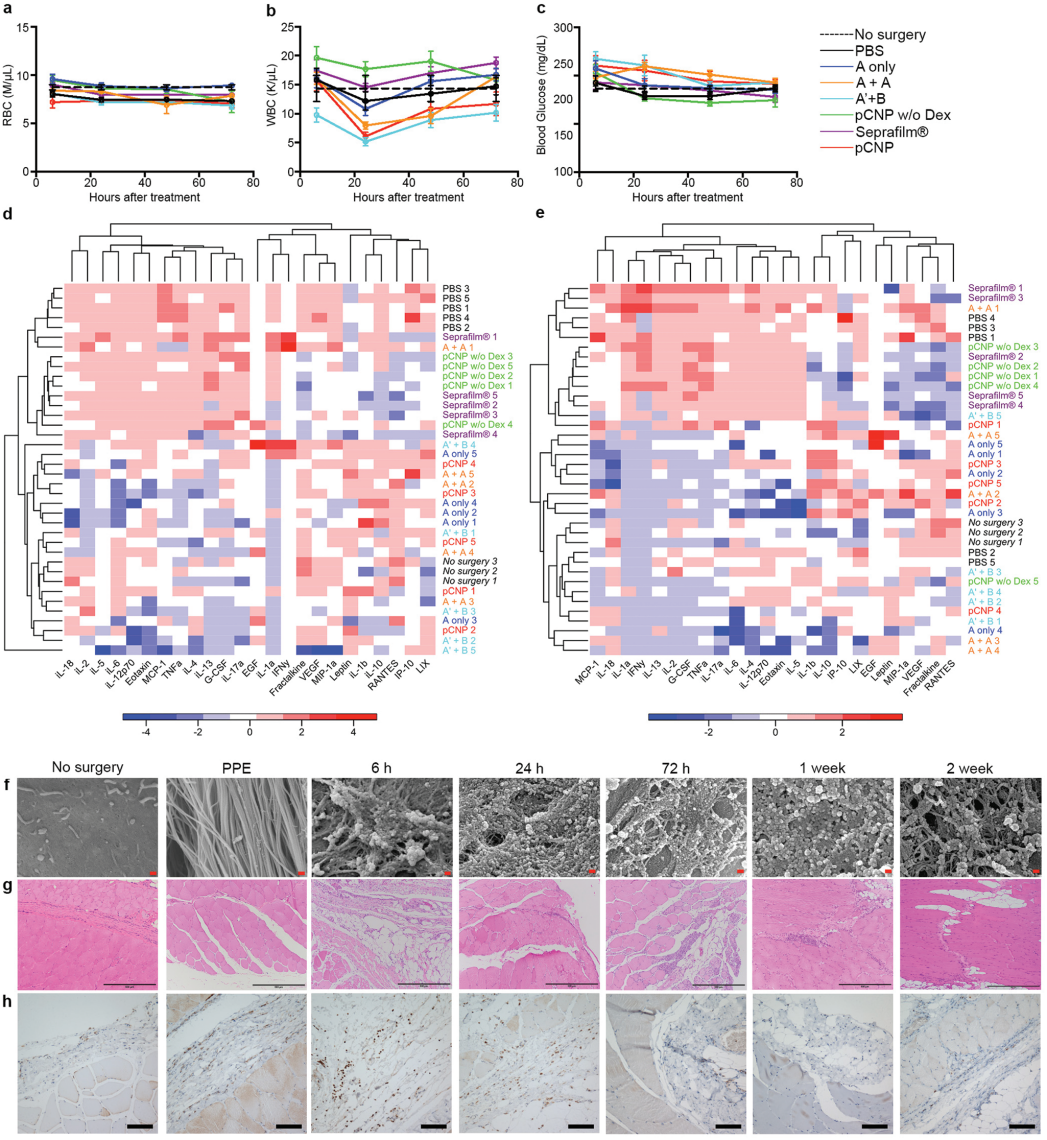
Toxicity and mechanism of pCNP in preventing postsurgical peritoneal adhesion in parietal peritoneal excision (PPE) model in rats. a–c) Blood test at 6, 24, 48, and 72 h after treatments: red blood cell count (a), white blood cell count (b), blood glucose concentration (c). Data represents mean ± standard error of the mean (SEM) (For A+A, *n* = 6; For pCNP w/o Dex, *n* = 7; For Seprafilm, *n* = 8; For other groups, *n* = 9). d,e) Heatmap showing serum cytokine/chemokine levels in each sample across all treatment groups after 24 h d) and 72 h e). Samples (*y* axis) and cytokine/chemokine levels (*x* axis) were heterarchical clustered. Color scale reflects cytokine/chemokine expression magnitude (red: high, blue: low). f) FESEM images showing the retention and biodegradation of pCNP on rats' abdominal wall at 6 h, 24 h, 72 h, 1 week, and 2 weeks after surgery and subsequent treatment with pCNP. Scale bar = 200 nm. g,h) Hematoxylin and eosin (H&E) staining images (g) and CD45 immunohistochemistry (IHC) staining images (h) showing the local inflammation on rats' abdominal wall at 6 h, 24 h, 72 h, 1 week, and 2 weeks after surgery and subsequent treatment with pCNP. For (g), scale bar = 500 µm; For (h), scale bar = 100 µm.

As inflammatory level plays an important role in adhesion formation, we assessed cytokines/chemokines in serum 24 and 72 h postsurgery with different treatments using immunology multiplex assay (Figure 4d,e, Figure S16, Supporting Information). Hierarchical clustering showed that PBS, pCNP w/o Dex, and Seprafilm groups clustered together and showed high expression of inflammatory markers such as interleukin family, TNFα, IFNγ, G‐CSF, and MCP‐1. On the contrary, pCNP group showed similar inflammatory level to no surgery group, suggesting a decreased level of inflammation during postsurgical healing process prevented adhesion formation.

To further understand the functions of pCNP during the healing process, we treated rats bearing PPE with pCNP and acquired their injured abdominal wall after 6 h, 24 h, 72 h, 1 week, and 2 weeks. FESEM images showed that collagen fibers were exposed in PPE model and most part of injured surface was recovered after 2 weeks (Figure S17, Supporting Information). Briefly, our PPE model caused injuries on the muscle layer. During the healing process, an initial inflammation was observed within 72 h with the emergence of some infiltrated macrophages and neutrophil. The injured area was made up by a large amount of collagens from 24 h to 1 week. During this time, small vessels and immature cells came out. After 2 weeks, the injured area was rebuilt by being filled up with fresh muscle cells. A Masson's trichrome stain was provided to show the damage and healing of muscle fibers during the period (Figure S18, Supporting Information). By magnifying the surface, we observed a dense NP layer formed and retained on collagen fibers for the whole process (Figure 4f, Figure S19, Supporting Information). We also found a decrease in NP density after 2 weeks, indicating the biodegradation of pCNP. We further imaged the live rats treated by pCNP with NIR fluorescence from 6 h to day 14 after surgery. We found that the fluorescent pCNP successfully targeted to the injured abdominal wall after surgery and kept there for 2 weeks with sustained decrease in fluorescent signal (Figure S20, Supporting Information). Hematoxylin and eosin (H&E) staining showed that mild inflammation occurred at 6 h mainly due to the surgery, and no severe active inflammation (immune infiltration) in other experimental arms (Figure 4g, Figure S21, Supporting Information). The local inflammatory degree was further confirmed by CD45 and IL‐1β immunohistochemistry (IHC), which consisted with H&E staining results (Figure 4h, Figures S22 and S23, Supporting Information). This suggests that pCNP was biocompatible and did not cause any severe inflammation in the tissue where they were administered.

Postsurgical adhesion is a common complication from surgical treatment. Despite its prevalence, there has been limited advance in its treatment. This is largely due to the difficulty of providing barriers over all injured epithelial/mesothelial surfaces. In addition, medical therapies such as anti‐inflammatories have systemic side effects that limit their use. Recent progress in material science shows that polymeric films can be used to improve the efficacy in preventing peritoneal adhesion.^28^, ^29^, ^30^ To overcome these challenges, we aimed to develop a biologically targeted barrier system that can also incorporate medical therapies to prevent postsurgical adhesions. Our technology exploits the advantages of targeted‐dual‐nanoparticle system with opposite surface charge, which forms a layer exactly on the injured site with satisfied density, at the same time showing better release kinetics for anti‐inflammatory drugs. Unlike traditional nanoparticles that lack the ability to target the injured surface and lower the layer density by steric and ionic hindrance, our pCNP can target the injured site with a basement membrane targeting ligand and fully cover it by electrostatic interaction and photo‐induced crosslinking. Unlike hydrogel‐like materials that do not always stay in place post surgery and have unsatisfactory release profile for the anti‐inflammatory drugs, our pCNP can retain on the injured site and release anti‐inflammatory drugs under a controlled manner during the whole healing process. Compared to Seprafilm that needs to avoid any contact from other organs as it is quite sticky to moist surface, our pCNP can be easily incubated or sprayed on an injured surface. The flexibility of UV exposure is easily achieved through the UV guidance system.

To achieve targeting, we utilized peptides that bind to collagen as targeting ligands. Since collagen/basement membranes are only exposed when epithelium/mesothelium is injured, our collagen‐targeted NPs will only bind to areas of tissue injury (denuded epithelium and mesothelium). Using in vitro and in vivo studies, we demonstrated our biological targeting is highly specific. To further improve the pCNP, we incorporated dexamethasone into the system to reduce inflammation and promote normal healing. As seen in our in vivo studies, the addition of dexamethasone indeed further reduced adhesion formation.

A key challenge in this study relates to the fact that adhesion formation is both qualitative and quantitative. To fully capture both measures of adhesions, we utilized a well‐established adhesion scoring system to analyze our in vivo data. To avoid bias, we blinded the surgeons during this experiment. Moreover, we have included the raw images with this work to enable unbiased interpretation.

We also want to note that pCNP is shown to be safe in our studies. Given the materials for pCNP are generally regarded as safe (GRAS) materials, we believe this technology is readily translated into clinical practice. To facilitate the translation, our future studies will examine crosslinking strategies that are faster and more convenient. Moreover, we will study whether medical therapies can be incorporated to further prevent adhesion formation.

In summary, we report the first biologically targeted approach to prevent postsurgical adhesions. We combined a collagen IV‐targeted NP with a photo‐crosslinkable NP to form a dense barrier over injured epithelial/mesothelial surfaces in a biologically targeted fashion. The barrier (pCNP) is also capable of delivering anti‐inflammatory therapeutics to further prevent adhesion formation. We demonstrated that pCNP can effectively prevent surgical adhesions in vivo, better than the commercial adhesion barrier Seprafilm, using a rat PPE model. Moreover, the pCNP did not show any significantly toxicity. This technology holds high potential for clinical translation, and its success will improve quality of life of surgical patients as well as reduce healthcare costs. Our study also demonstrates the potential applications of biologically targeted nanomaterials.

## Experimental Section

*Materials*: Methoxy‐poly(ethylene glycol)‐poly(lactic‐*co*‐glycolic acid) block copolymer (mPEG‐PLGA) (AK029; LA:GA = 50:50 (w:w); MW: ≈3000:36000 Da), poly(lactic‐*co*‐glycolic acid)‐ poly(ethylene glycol)‐maleimide block copolymer (PLGA‐PEG‐Mal) (AI110; MW: ≈30000–5000 Da), and poly(lactic‐*co*‐glycolic acid)‐ poly(ethylene glycol)‐ carboxylic acid block copolymer (PLGA‐PEG‐COOH) (AI034; MW: ≈3400:17000 Da) were obtained from Polyscitech. Collagen IV‐targeting peptide with an amino acid sequence of KLWVLPKGGGC was purchased from UNC High‐Throughput Peptide Synthesis and Array Facility. The peptide was synthesized via automated Fmoc solid phase peptide synthesis method and purified by high‐performance liquid chromatography (HPLC). Peptide homogeneity was confirmed by MALDI‐TOF mass spectroscopy and analytical HPLC. Dexamethasone 21‐Palmitate was obtained from Toronto Research Chemicals. Polyethyleneimine (M.N. 60000, 50 wt% aq. solution, branched), acetone, dimethylformamide (DMF) anhydrous, methanol (MeOH), and water (HPLC grade) were obtained from Fisher Scientific. Sulfosuccinimidyl 6‐(4,4′‐azipentanamido) hexanoate (sulfo‐LC‐SDA), EDC/sulfo‐NHS, and triethylamine, were obtained from Sigma‐Aldrich. Seprafilm was obtained from Sanofi Biosurgery.

*pCNP Preparation*: pCNP was prepared separately. NP‐A was fabricated through nanoprecipitation technique.^31^, ^32^ Collagen IV‐targeting peptide‐functionalized poly(ethylene glycol)‐poly(lactic‐*co*‐glycolic acid) (Col‐PEG‐PLGA) was synthesized first according to previous reports.^16^, ^18^ Briefly, maleimide functionalized PEG‐PLGA (Mal‐PEG‐PLGA) and collagen IV‐targeting peptide (KLWVLPKGGGC‐NH2) were dissolved in 5 mL anhydrous DMF with a molar ratio of 1:1.2. Triethylamine (5 µL) was added and the reaction was stirred under nitrogen at room temperature for 24 h. The solution was precipitated in cold MeOH and was centrifuged at 3000 g for 10 min. The pellet was washed with MeOH twice and dried under vacuum. To prepare NP‐A, Col‐PEG‐PLGA and PEG‐PLGA (1:4 weight ratio) were dissolved into acetone with a final polymer concentration of 10 mg mL^−1^. Dex‐Pal (5% wt of total polymer) was added into the solution. The organic phase was added dropwise into aqueous phase (endotoxin free H_2_O) through a syringe under the oil to water ratio of 1:2. The solution was stirred at room temperature under a vacuum until the acetone completely evaporated. The solution were centrifuged and washed with endotoxin free H_2_O. To prepare NP‐B, NP‐COOH was first prepared through a similar nanoprecipitation technique by using COOH‐PEG‐PLGA and PEG‐PLGA (1:1 weight ratio).^33^ The NP‐COOH (1 mg mL^−1^) were reacted with EDC/sulfo‐NHS in PBS for 10 min, followed by adding branched PEI with a concentration of 2 mg mL^−1^ for a final NP to PEI weight ratio of 5:4. Nanoparticles with PEI layer (NP‐PEI) was then washed twice and collected. Next, NP‐PEI was stirred with sulfo‐LC‐SDA (3:1 by weight) in PBS for half an hour at room temperature. The nanoparticles were finally harvested after washing twice with endotoxin free H_2_O.

*pCNP Characterization*: pCNP was characterized by intensity‐average diameter (*D*_h_, also known as hydrodynamic diameter) and mean zeta potential (mean ζ) using Zetasizer Nano ZS Instrument (Malvern, Inc.). All measurements were based on the average of three separate measurements.

The drug loading of Dex‐Pal in NP‐A (weight of Dex‐Pal divided by weight of NP‐A) was tested by HPLC. A known amount of freeze‐dried NP‐A was dissolved in 1 mL DCM. After evaporating the DCM, 1 mL of 50% acetonitrile (ACN) in water was added to dissolve the extracted drugs. The solution was then filtered by 0.45 mm PVDF membrane for HPLC analysis. The column effluent was detected at 236 nm with a continuous gradient mobile phase from 50% to 100% ACN in water over 20 min. All values were based on the average of three separate replicate measurements.

*In Vitro Analysis for pCNP*: A collagen‐IV‐coated glass cover was prepared for in vitro analysis by incubating a poly(D‐lysine) (PDL)‐coated glass cover slip (Neuvitro, GG‐12‐1.5‐PDL) in a 100 µg mL^−1^ solution of collagen IV overnight at room temperature. The collagen‐IV‐coated glass cover was washed twice before use.

10 mg mL^−1^ NP with (NP‐A) or without (NP‐A′) collagen IV‐targeting ligand was incubated with collagen‐IV‐coated or noncoated glass covers for 10 min at room temperature. The glass covers were then washed twice with saline and water. Next, 10 mg mL^−1^ NP‐B was incubated with the glass covers for 10 min under UV irradiation at 365 nm and 50 mW cm^−2^. Then, the glass covers were washed with saline and water, and left to air dry at room temperature. The NP layer formation was checked by Zeiss Supra 25 field emission scanning electron microscope.

For in vitro drug release profile, pCNP was first formed on collagen‐IV‐coated glass covers as is mentioned above. The glass covers were placed in PBS with or without esterase (T lanuginosus lipase, Sigma) at 100 U mL^−1^ and shaken at 37 °C. The release buffer was substituted at 6 h, 24 h, 48 h, 72 h, 120 h, 9 days, and 14 days and freeze‐dried for quantitative analysis. Cumulative release of Dex‐Pal and its active form dexamethasone were performed with a Shimadzu SPD‐M20A high‐performance liquid chromatography (HPLC) equipped with a diode array detector and a C18 25 cm × 4.6 mm, 5 µm column (Supelco, Sigma). Samples were eluted using a gradient binary solvent system from 50% acetonitrile in water to 100% for Dex‐Pal and 10%–80% for dexamethasone at a flow rate of 1 mL min^−1^. Dex‐Pal and dexamethasone elution were monitored at 236 and 240 nm, respectively. The experiment was repeated three times.

For in vitro cytotoxicity analysis, 96‐well plate was coated with collagen IV and seeded with NIH/3T3 murine fibroblast cells at 10 000/well for 2 and 24 h, 5000/well for 72 h. NP‐A with or without Dex‐Pal was incubated for 10 min and washed twice with saline. NP‐B was incubated for 10 min with or without UV irradiation at 365 nm and 50 mW cm^−2^. The plate was then washed twice with saline and added cell culture medium at 100 µL per well. Different NP concentrations at 5, 10, and 20 mg mL^−1^ were used for both NP‐A and NP‐B. After 2, 24, and 72 h, the plate was tested with MTS assay (CellTiter 96 AQueous One Solution Cell Proliferation Assay, Promega).

*Photo‐DSC Analysis*: Cross‐linking kinetics were determined by photocalorimetry using a Discover DSC with the PCA accessory, equipped with a Omnicure S‐2000 mercury UV light source with a 365 nm external filter (TA instruments, New Castle, DE, USA). 20 µL of the nanoparticle solution was added to an aluminum DSC sample pan without a lid and placed in the DSC cell, which was held at a constant temperature of 5 °C under a 10 mL min^−1^ nitrogen flow. After a 2.5 min isothermal step, samples were exposed to UV light for 20 min at 50 mW cm^−2^. Enthalpy was calculated by integrating the normalized heat flow curve using a horizontal baseline at the heat flow value after 20 min of exposure.

*Sample Size Calculations and Analysis for In Vivo Efficacy Studies*: Sample size is calculated based on the preliminary data. An effect size of 1.821 was calculated. The nonparametric analog of this effect size can be stated in terms of p1 = Pr (X < Y), or an observation in Group X is less than an observation in Group Y when H1 is true. The null hypothesis being tested is p1 = 0.5. For effect size 1.821, p1 = 0.099. A sample size of 8 in each group will have 80% power to detect a probability of 0.099 that an observation in Group X is less than an observation in Group Y, using a Wilcoxon (Mann‐Whitney) rank‐sum test, with a 0.05 two‐sided significance level.

*Animal Model*: The in vivo analysis utilized parietal peritoneum excision (PPE) to generate adhesion on rat (Figure S2, Supporting Information).^25^ Briefly, a survival–surgery was carried out on Sprague–Dawley rats in which a ≈2 cm × 5 cm patch of peritoneum and the underlying muscle layer was excised from the left abdominal wall remote from the midline laparotomy. The wound was washed with saline and closed. After 14 d, a second‐look laparotomy was performed to assess adhesion formation according to the scoring systems described in the literature.^25^, ^26^, ^27^ Briefly, a four‐point scale was performed for qualitative assessment of adhesion where 0 = no adhesions; 1 = filmy adhesions; 2 = moderate‐thickness adhesion; 3 = dense‐thickness adhesion. A five‐point scale was performed for quantitative assessment of adhesion where the percentage of adhesion area to the surgical area was quantified as 0 = 0% adhesions; 1 = less than 25%; 2 = 25–49%; 3 = 50–74%; 4 = 75–100% adhesions.

*Treatment of Injured Surface with pCNP on Rats*: Sprague–Dawley rats at 25–45 weeks of age and 400–600 g of body weight underwent the aforementioned survival surgery. Prior to closure, rats were laid on the side ipsilateral to the excision to form a pocket‐like cavity for NP incubation (Figure S3, Supporting Information). NP‐A (2 mL) at 10 mg mL^−1^ was first administered to the peritoneal cavity and allowed to incubate for 10 min. The injured surface was washed with saline twice. NP‐B (2 mL) at 10 mg mL^−1^ was then administered to the peritoneal cavity and the injury site was concurrently irradiated for 10 min with 365 nm UV light at an intensity of 50 mW cm^−2^ from light guides, which were fitted to an Omnicure S‐2000 light source as used in the photo‐DSC apparatus. The peritoneal cavity was washed twice with saline after incubation and the wound was closed. The rats were under constant monitoring and blood was collected at 6, 24, 48, and 72 h after surgery for analysis. 100 µL whole blood was collected for complete blood count test and 20 µL serum was collected for blood glucose test. After 14 d, rats were euthanized by CO_2_. The abdomen was opened via a right lateral U‐shaped laparotomy to prevent the disturbance of adhesion area. The lateral with treatment and the other lateral without any manipulation were recorded by optical photos. Two experimenters, blinded to the procedure, assessed all the adhesions according to the aforementioned scoring systems. The related tissues including muscle, skin, and peritoneal adhesion were excised and fixed in 10% neutral buffered formalin for histological analysis.

*Histological Analysis*: Tissues were fixed in 10% neutral buffered formalin at room temperature for ≈3 d. Fixed tissues were processed on a Leica ASP 6025 tissue processor, embedded in paraffin wax, and sectioned at 4 µm thickness on a Leica RM2245 microtome and mounted on VWR Superfrost Plus microscope slides. Tissue sections were H&E stained using Richard–Allen Hematoxylin 2 and Eosin Y and cover‐slipped. Tissue sections were Masson's trichrome stained using standard staining protocol from Thermo Scientific. Histologic changes were evaluated by a board‐certified veterinary pathologist.

*Live Rats Imaging*: NIR fluorescent pCNP was synthesized by adding PLGA‐FPI749 (Polyscitech, AV028) to NP‐A (40 wt%) and NP‐B (25 wt%). Rats were excised a ≈2 cm × 2 cm patch of peritoneum with the underlying muscle layer from the left abdominal wall and treated with PBS or NIR fluorescent pCNP. Rats were imaged by IVIS Kinetic from 6 h to day 14 after surgery and treatments.

*Immunohistochemical Analysis*: Immunohistochemical analysis was performed on paraffin slides using anticollagen I antibody (34 710, Abcam), anticollagen IV antibody (6586, Abcam), antifibronectin antibody (23 751, Abcam), anti‐CD45 antibody (10 558, Abcam), and anti‐IL‐1β antibody (ab9722, Abcam). Antigen retrieval was performed using Ventana's CC2 (pH 6.0) for 8 min at 90 °C for anticollagen I; Ventana's CC2 (pH 6.0) for 40 min at 100 °C for anticollagen IV; Ventana's CC1 (pH 8.5) for 8 min at 90 °C for antifibronectin; and Ventana's CC2 (pH 6.0) for 40 min at 90 °C for anti‐CD45. The slides were given a hydrogen peroxide block for 32 min and then incubated in a blocking reagent (Rodent Block R, RBR962G, Biocare) for 1 h at room temperature. The primary antibody was added at 1:100 for anticollagen I and 1:50 for the others using Discovery Ab Diluent, 760‐108, followed by the secondary antibody (Ventana Omap OmniMap anti‐Rb‐HRP, 760‐4311, ready to use) for 32 min at room temperature. The slides were then treated with DAB and counterstained with Hematoxylin II for 12 min and then Bluing Reagent for 4 min.

*Time‐Point Tissue Analysis*: Rats bearing PPE surgery were treated by pCNP. The injured tissues were taken after 6 h, 24 h, 72 h, 1 week, and 2 weeks of the treatment, rinsed briefly with PBS to remove surface debris, followed by immersion fixation in 10% neutral buffered formalin for histological analysis, immunohistochemical analysis, and FESEM. To prepare FESEM samples, after initial fixation for several hours to overnight in formalin, the region of interest was dissected out and placed in 2% paraformaldehyde/2.5% glutaraldehyde/0.15 m sodium phosphate buffer, pH 7.4. Specimens were stored in the fixative overnight to several days at 4 °C before processing for SEM. After three washes with 0.15 m sodium phosphate buffer, pH 7.4 (PB), the samples were postfixed in 1% osmium tetroxide in PB for 1 h followed by three 30 min washes in deionized water. The samples were dehydrated in a grade series of ethanol, transferred to a Samdri‐795 critical point dryer, and dried using carbon dioxide as the transitional solvent (Tousimis Research Corporation, Rockville, MD, USA). Tissues were mounted on aluminum planchets using silver paste and coated with 15 nm of gold–palladium alloy (60Au:40Pd, Hummer X Sputter Coater, Anatech USA, Union City, CA, USA). Images were taken using a Zeiss Supra 25 FESEM operating at 5 kV, using the SE2 detector, 30 µm aperture, and approximate working distance of 10 to 12 mm (Carl Zeiss Microscopy, LLC, Peabody, MA, USA).

*Analysis of Postsurgery Inflammatory Levels*: Rats bearing PPE surgery were treated with different experimental arms. Serum was collected at 24 and 72 h after surgery with SST Serum Separation Tubes (BD Vacutainer Venous Blood Collection Tubes). Immunology multiplex assay was performed with milliplex map rat cytokine/chemokine magnetic bead panel (RECYMAG65K27PMX, Millipore). To examine and visualize inflammatory levels after different treatments, each cytokine/chemokine level was log2 transformed and standardized. Hierarchical clustering with Euclidean distance and complete linkage was performed and heatmap was used for visualization. Pearson correlations among samples were also calculated and plotted. All clustering and heatmap analysis were done under R version 3.5.1 using package gplots (heatmap.2).

## Conflict of Interest

The authors declare no conflict of interest.

## Supporting information

Supplementary
