# Supplementary material for: Biologically Targeted Photo‐Crosslinkable Nanopatch to Prevent Postsurgical Peritoneal Adhesion
Source: Adv Sci (Weinh). 2019 Aug 13;6(19):1900809. doi: 10.1002/advs.201900809 (PMC6774057; doi:10.1002/advs.201900809)
Supplement: Supplementary file 1 — Supplementary [file ADVS-6-1900809-s001.pdf]

Copyright WILEY-VCH Verlag GmbH & Co. KGaA, 69469 Weinheim, Germany, 2019.

ADVANCED SCIENCE
Open Access

Supporting Information

for *Adv. Sci.,* DOI: 10.1002/advs.201900809

Biologically Targeted Photo-Crosslinkable Nanopatch to Prevent Postsurgical Peritoneal Adhesion

*Yu Mi, Feifei Yang, Cameron Bloomquist, Youli Xia, Bo Sun, Yanfei Qi, Kyle Wagner, Stephanie A. Montgomery, Tian Zhang, and Andrew Z. Wang**

<u>**Supplementary Information**</u>

Biologically targeted photo-crosslinkable nano-patch to prevent postsurgical peritoneal adhesion

Yu Mi, Feifei Yang, Cameron Bloomquist, Youli Xia, Bo Sun, Yanfei Qi, Kyle Wagner, Stephanie Montgomery, Tian Zhang, Andrew Z. Wang[*]

Dr. Yu Mi, Dr. Feifei Yang, Dr. Cameron Bloomquist, Dr. Bo Sun, Dr. Yanfei Qi, Dr. Kyle Wagner, Prof. Andrew Z. Wang

Laboratory of Nano- and Translational Medicine, Carolina Center for Cancer Nanotechnology Excellence, Carolina Institute of Nanomedicine, Lineberger Comprehensive Cancer Center, Department of Radiation Oncology, University of North Carolina at Chapel Hill, Chapel Hill, North Carolina 27599, United States.

Email: zawang@med.unc.edu

Dr. Cameron Bloomquist

School of Pharmacy, University of North Carolina at Chapel Hill, Chapel Hill, North Carolina 27599, United States.

Youli Xia

Department of Genetics, University of North Carolina at Chapel Hill, Chapel Hill, North Carolina 27599, United States.

Prof. Stephanie Montgomery

Department of Pathology and Laboratory Medicine, University of North Carolina at Chapel Hill, Chapel Hill, North Carolina 27599, United States.

Prof. Tian Zhang

Department of Medical Oncology, Department of Medicine, Duke University Medical Center, Durham, North Carolina 27710, United States.

Dr. Feifei Yang

Institute of Medicinal Plant Development (IMPLAD), Chinese Academy of Medical Sciences & Peking Union Medical College, Haidian District, Beijing, P.R. China.

Dr. Yanfei Qi

School of Public Health, Jilin University, Changchun, Jilin, P.R. China.

Table of Contents

Supplementary Fig. S1. Synthesis of collagen IV-targeting peptide functionalized PEG-PLGA (Col-PEG-PLGA) and associated 1H NMR.

Supplementary Fig. S2. pCNP formation from nanoparticles upon UV irradiation.

Supplementary Fig. S3. Repeated FESEM images showing the formation of a nano-patch on non-coated (**a**) and collagen IV-coated (**b**) glass cover slides using different approaches.

Supplementary Fig. S4. FESEM images showing the retention and degradation of pCNP on collagen IV-coated glass cover slides in PBS at 37 °C. Numbers showing the integrated density of gray value of the figures.

Supplementary Fig. S5. Release profile of dexamethasone (Dex) and dexamethasone 21-Palmitate (Dex-Pal) from pCNP on collagen IV-coated glass cover in PBS at 37 °C.

Supplementary Fig. S6. Cell viability of NIH/3T3 fibroblast cells after treating with UV irradiation and/or pCNP on collagen IV-coated 96-well plate.

Supplementary Fig. S7. Photos of parietal peritoneum excision (PPE) rat model showing injured area during abdominal surgery and adhesion after 14 days.

Supplementary Fig. S8. Representative immunohistochemistry (IHC) analysis of rat abdominal wall in parietal peritoneum excision (PPE) model. Scale bar = 200 µm.

Supplementary Fig. S9. a, Photos showing the UV light guide apparatus used during postsurgical treatment. b, Photos showing the surgery process and the administration of pCNP.

Supplementary Fig. S10. Postsurgical peritoneal adhesion on rats in each of the experimental groups 14 days after treatments.

Supplementary Fig. S11. Zoom in views of the postsurgical peritoneal adhesion on rats 14 days after treatments.

Supplementary Fig. S12. a-d, Representative H&E staining histology tissue images showing muscle and adhesion/fibrosis after abdominal surgery. For (a) and (b), scale bar = 2 mm; For (c) and (d), scale bar = 100 µm.

Supplementary Fig. S13. Representative H&E staining histology tissue images showing the adhesion/fibrosis after treatments. For PBS and A+A group, scale bar = 1 mm; for others, scale bar = 500 µm.

Supplementary Fig. S14. Representative Masson's trichrome staining histology tissue images.

Supplementary Fig. S15. Toxicity assessment of rats at 6 h, 24h, 48 h and 72 h after treatments.

Supplementary Fig. S16. A Pearson correlation matrix of each sample versus other samples (including itself as the diagonal line) demonstrates similarities of inflammation among different treatment groups.

Supplementary Fig. S17. FESEM images showing the healing process of rats' abdominal wall after PPE surgery and subsequent treatment with pCNP.

Supplementary Fig. S18. Representative Masson's trichrome staining histology tissue images on rats' abdominal wall at 6 h, 24 h, 72 h, 1 week and 2 weeks after surgery and subsequent treatment with pCNP.

Supplementary Fig. S19. Zoom out FESEM images showing the retention and biodegradation of pCNP on rats' abdominal wall at 6 h, 24 h, 72 h, 1 week and 2 weeks after surgery and subsequent treatment with pCNP.

Supplementary Fig. S20. a, IVIS imaging of rats from 6 h to day 14 after surgery and treatment.

Supplementary Fig. S21. Zoom out hematoxylin and eosin (H&E) staining images on rats' abdominal wall at 6 h, 24 h, 72 h, 1 week and 2 weeks after surgery and subsequent treatment with pCNP.

Supplementary Fig. S22. Zoom out CD45 immunohistochemistry (IHC) staining images of rats' abdominal wall at 6 h, 24 h, 72 h, 1 week and 2 weeks after surgery and subsequent treatment with pCNP. Scale bar = 200 μm.

Supplementary Fig. S23. IL-1β immunohistochemistry (IHC) staining images of rats' abdominal wall at 6 h, 24 h, 72 h, 1 week and 2 weeks after surgery and subsequent treatment with pCNP.

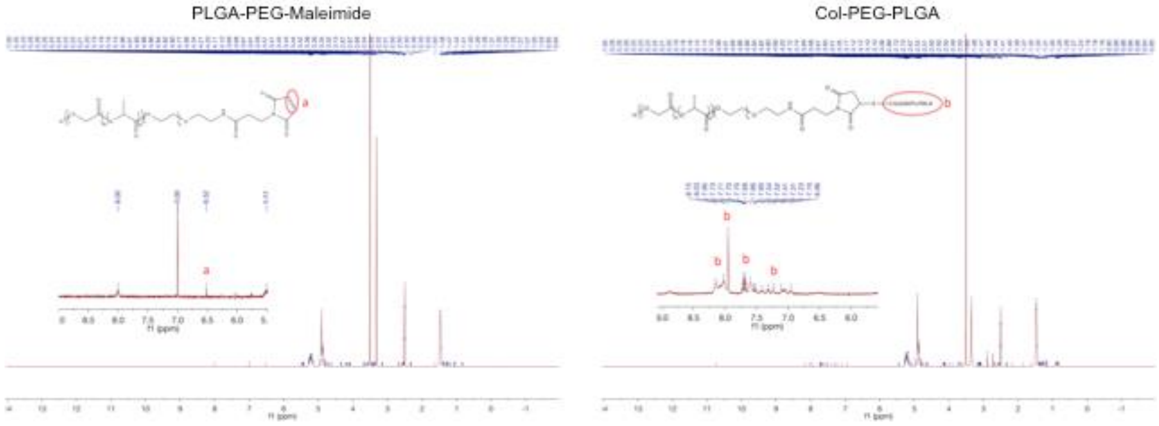

Supplementary Fig. S1. Synthesis of collagen IV-targeting peptide functionalized PEG-PLGA (Col-PEG-PLGA) and associated 1H NMR.

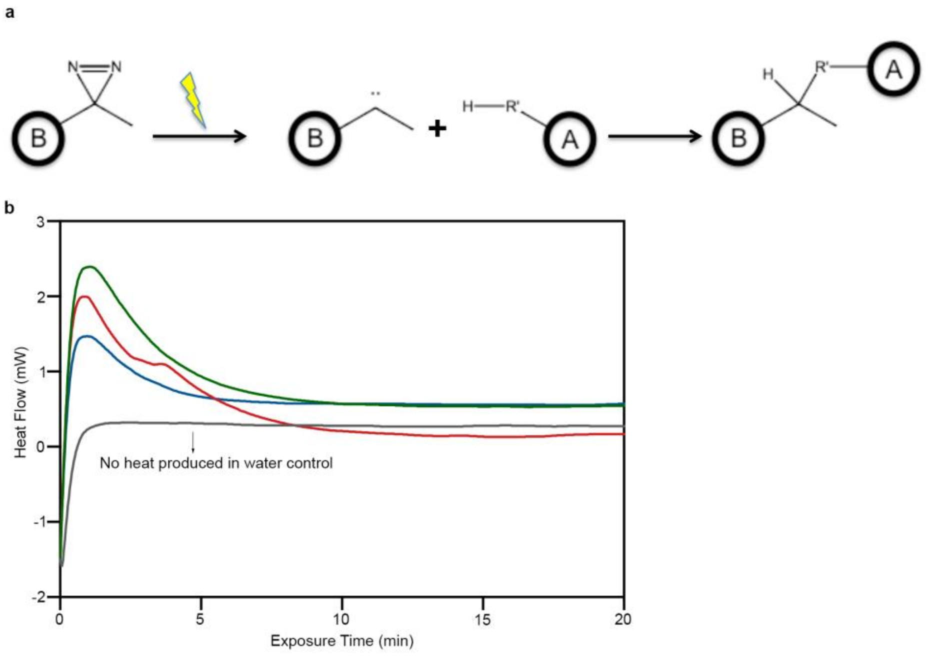

c

	UV intensity (mW/cm^2)	Solution (μL)	A (mg)	B (mg)	Ratio (B:A)	Enthalpy (J/g)
Black	25	20	0	0	0	0
Blue	25	20	0.15	1.71	11:1	8.4
Green	25	20	0.29	3.42	11:1	17.6
Red	25	20	0.17	4.1	24:1	21.8

Supplementary Fig. S2. pCNP formation from nanoparticles upon UV irradiation. **a,** Reaction schematic for the photo-induced crosslinking of nanoparticles via the diazirine functional group of NP-B. **b,** Photo-DSC analysis of the crosslinking reaction with different NP-A and NP-B concentrations. **c,** Calculated reaction enthalpy during diazirine crosslinking.

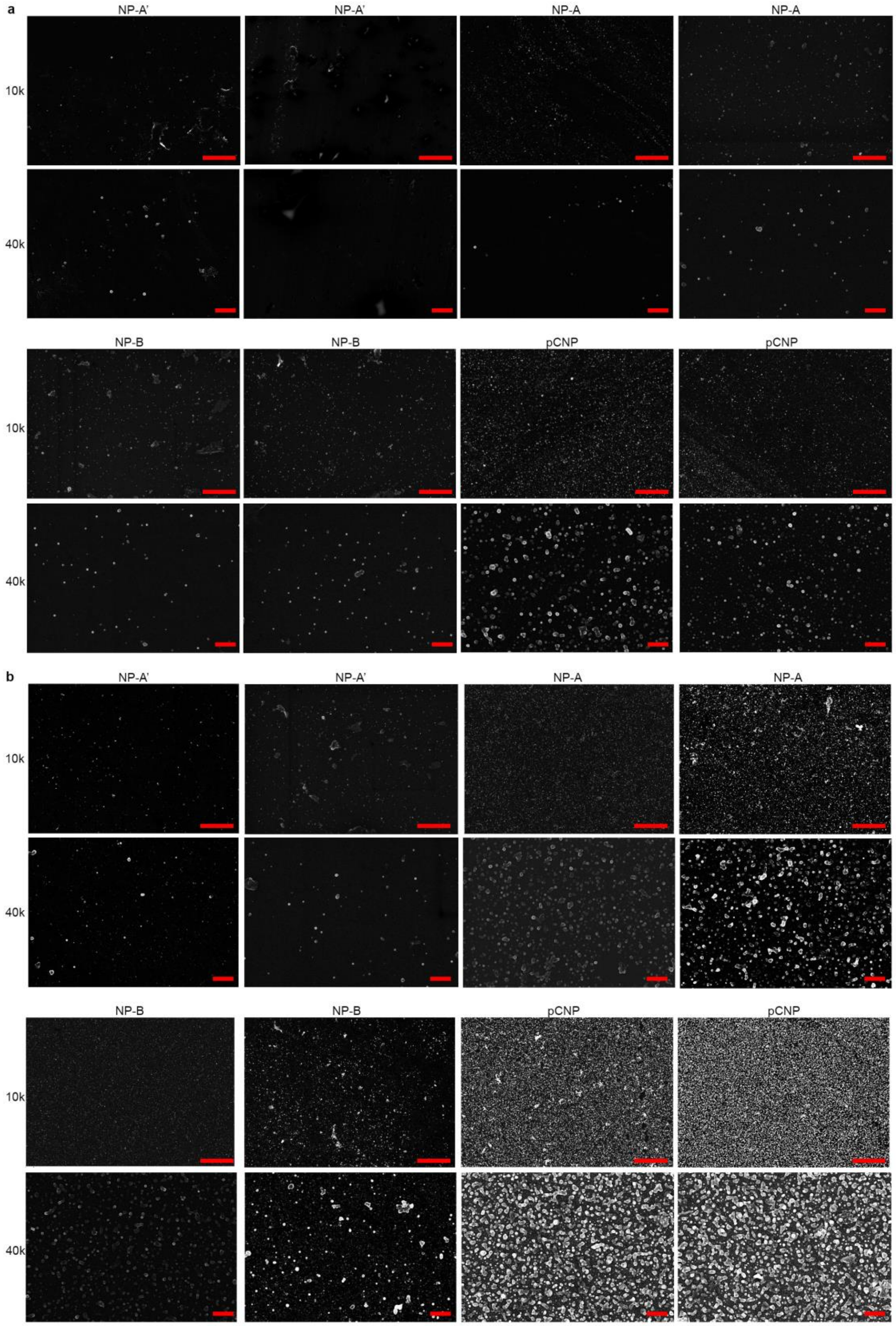

Supplementary Fig. S3. Repeated FESEM images showing the formation of a nano-patch on non-coated (**a**) and collagen IV-coated (**b**) glass cover slides using different approaches.

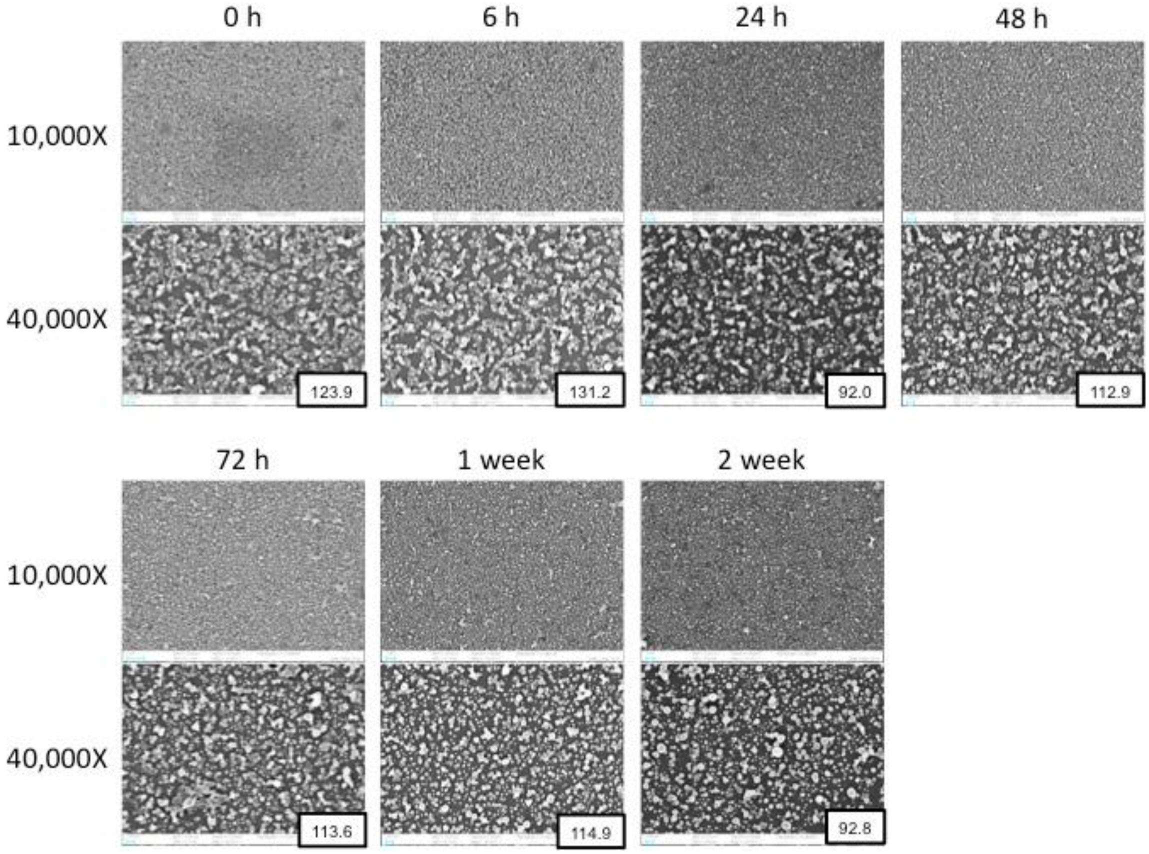

Supplementary Fig. S4. FESEM images showing the retention and degradation of pCNP on collagen IV-coated glass cover slides in PBS at 37 °C. Numbers showing the integrated density of gray value of the figures.

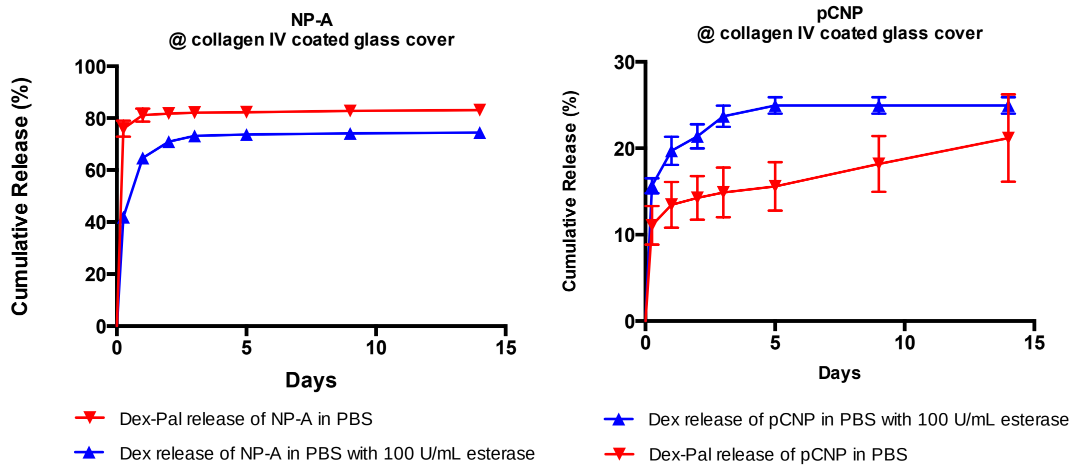

Supplementary Fig. S5. Release profile of dexamethasone (Dex) and dexamethasone 21-Palmitate (Dex-Pal) from NP-A and pCNP on collagen IV-coated glass cover in PBS at 37 °C.

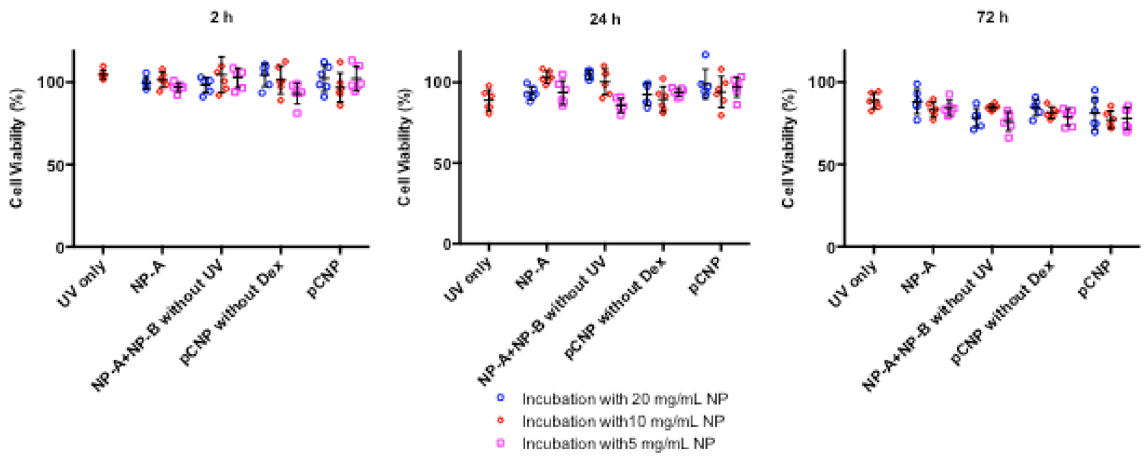

Supplementary Fig. S6. Cell viability of NIH/3T3 fibroblast cells after different treatments on collagen IV-coated 96-well plate.

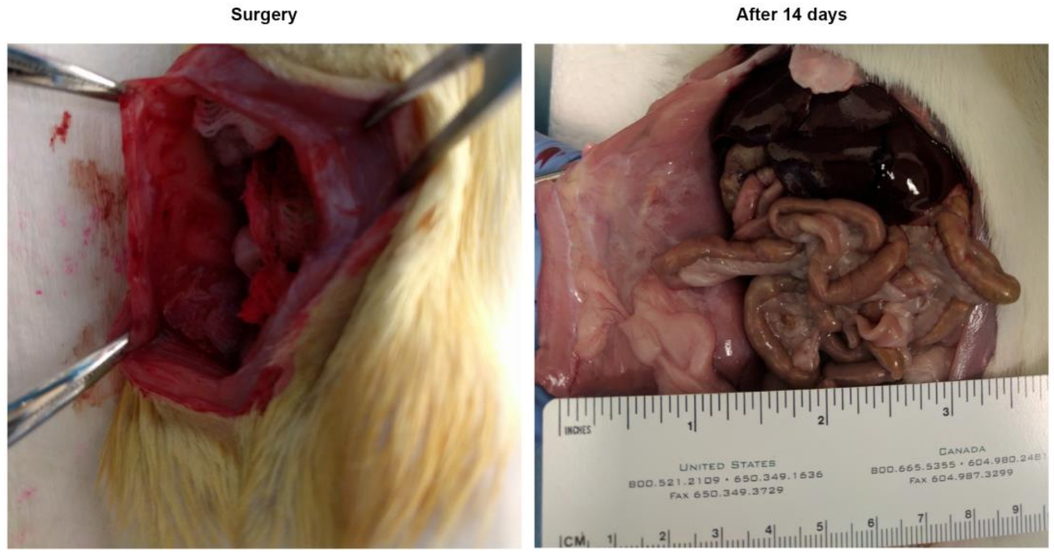

Supplementary Fig. S7. Photos of parietal peritoneum excision (PPE) rat model showing injured area during abdominal surgery and adhesion after 14 days.

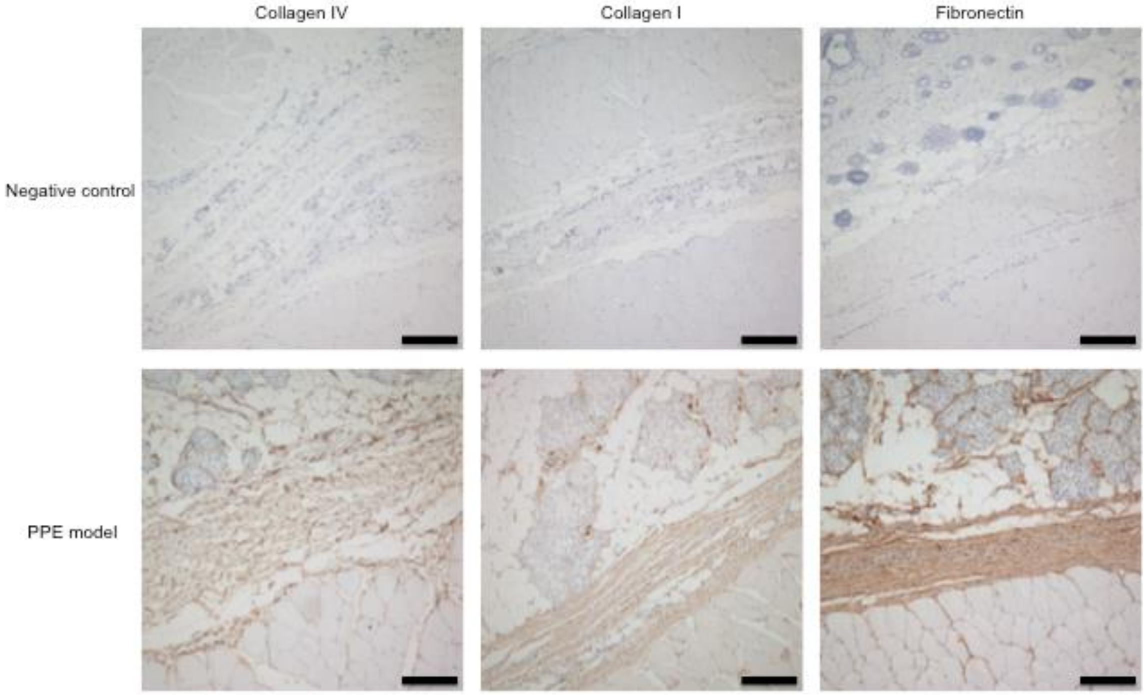

Supplementary Fig. S8. Representative immunohistochemistry (IHC) analysis of rat abdominal wall in parietal peritoneum excision (PPE) model. Scale bar = 200 μm.

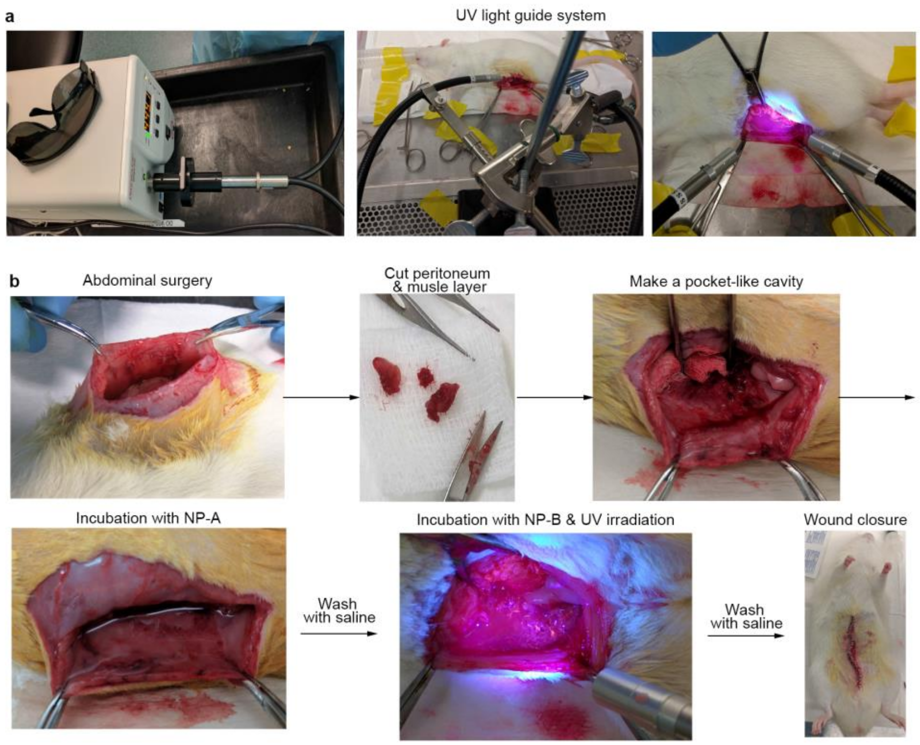

Supplementary Fig. S9. a, Photos showing the UV light guide apparatus used during postsurgical treatment. **b,** Photos showing the surgery process and the administration of pCNP.

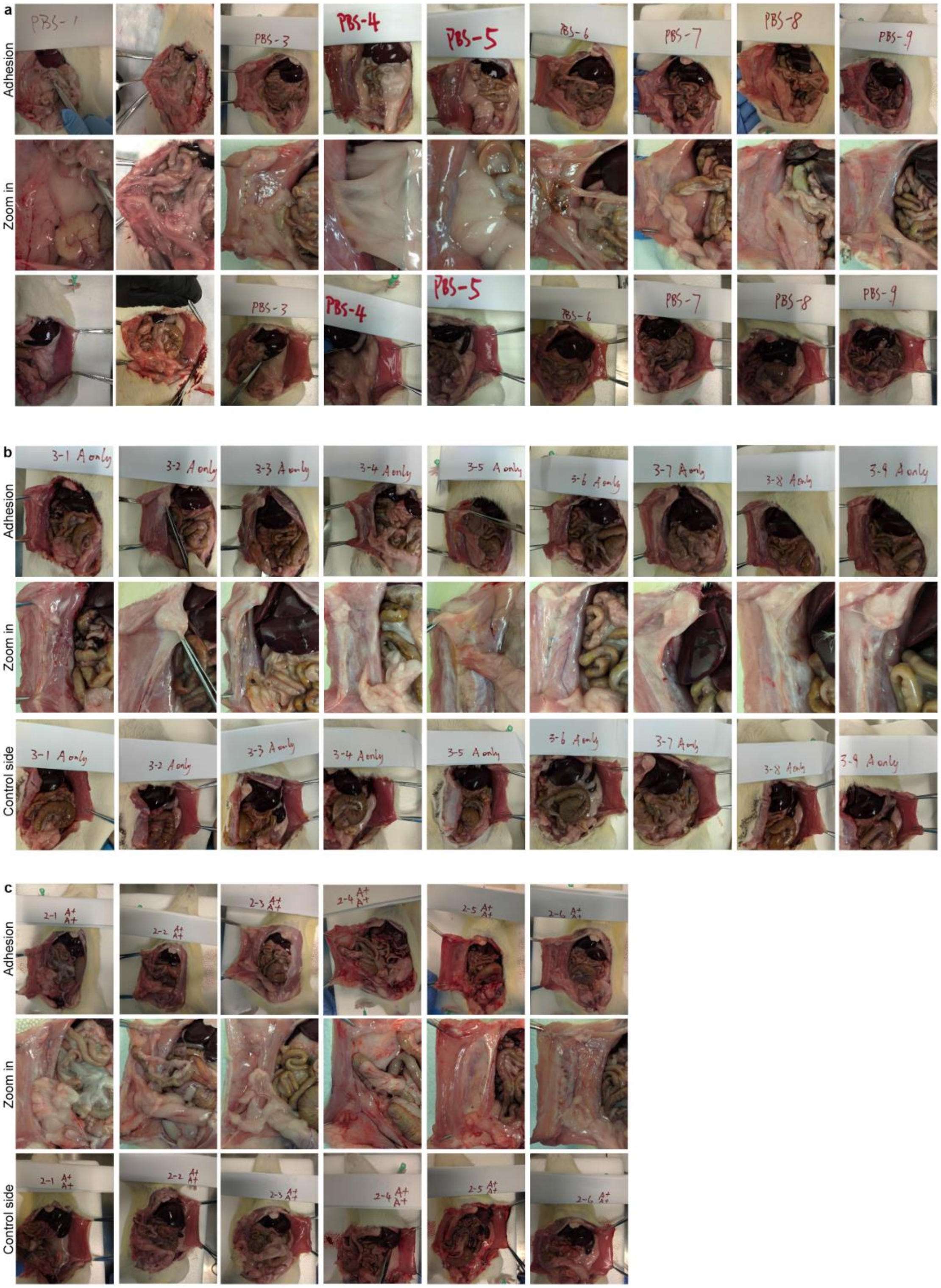

a
Adhesion
Zoom in
PBS-1
PBS-3
PBS-4
PBS-5
PBS-6
PBS-7
PBS-8
PBS-9
b
Adhesion
Zoom in
Control side
3-1 A only
3-2 A only
3-3 A only
3-4 A only
3-5 A only
3-6 A only
3-7 A only
3-8 A only
3-9 A only
c
Adhesion
Zoom in
Control side
2-1 A+ A+
2-2 A+ A+
2-3 A+ A+
2-4 A+ A+
2-5 A+ A+
2-6 A+ A+

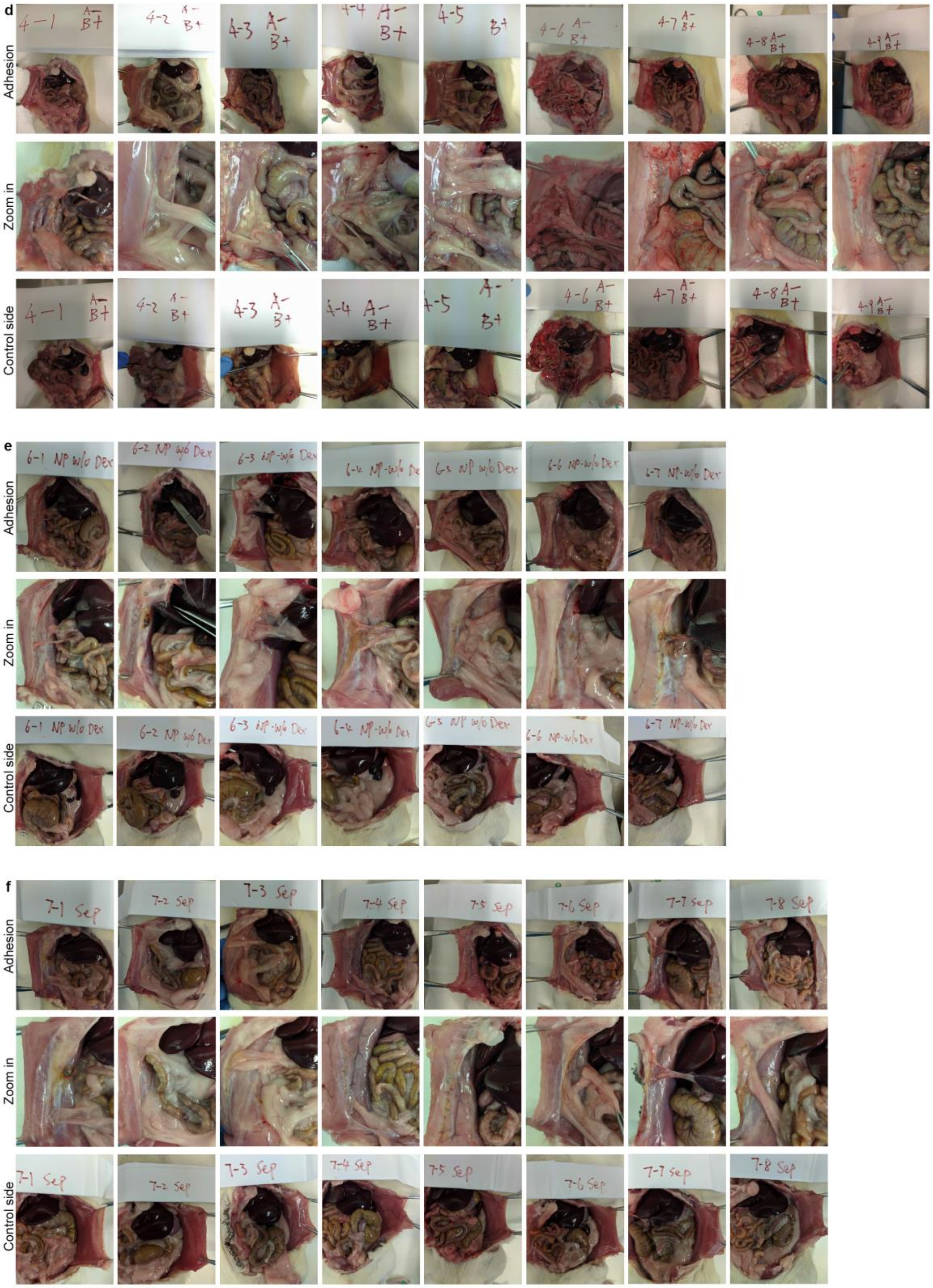

d
Adhesion
Zoom in
Control side
e
Adhesion
Zoom in
Control side
f
Adhesion
Zoom in
Control side

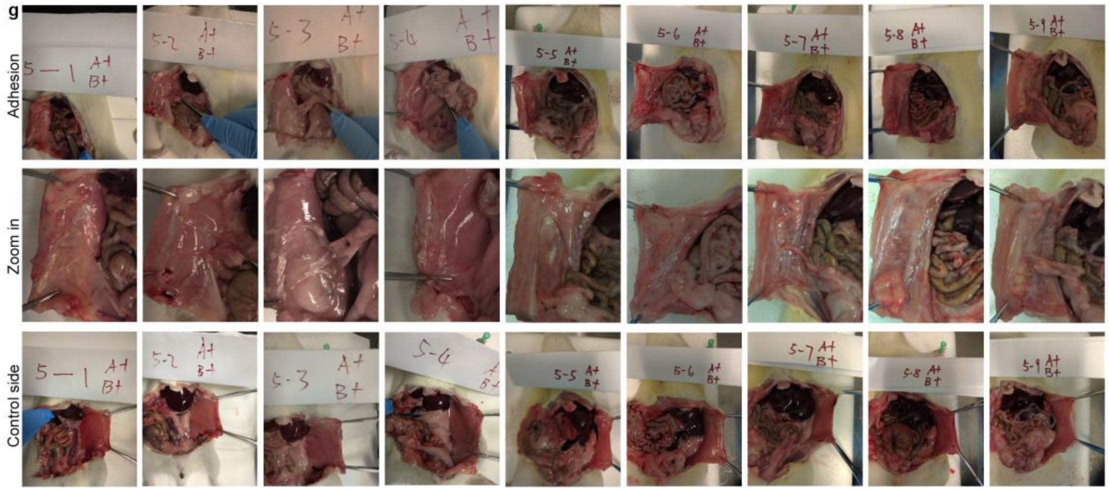

Supplementary Fig. S10. Postsurgical peritoneal adhesion on rats in each of the experimental groups 14 days after treatments.

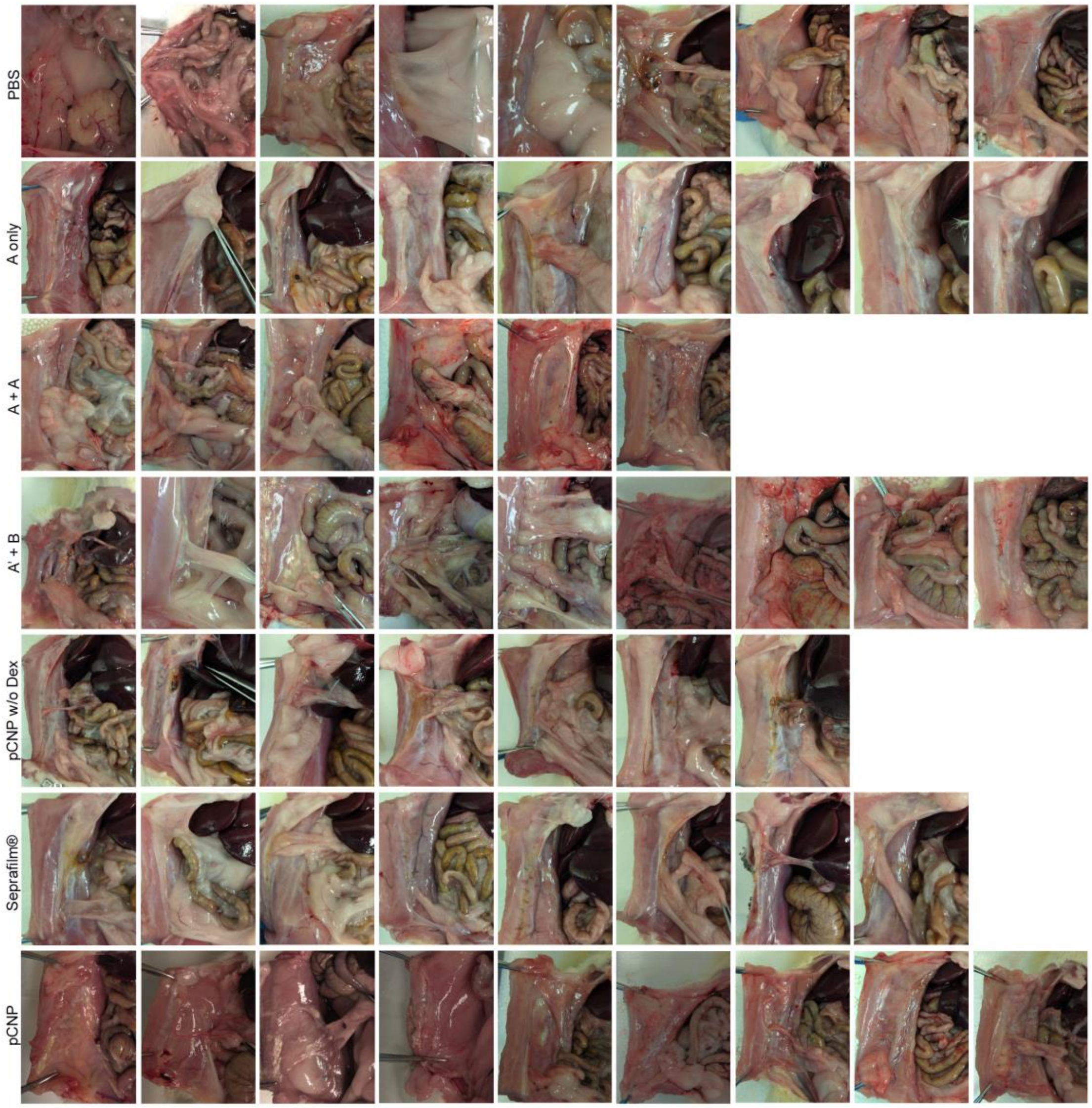

Supplementary Fig. S11. Zoom in views of the postsurgical peritoneal adhesion on rats 14 days after treatments.

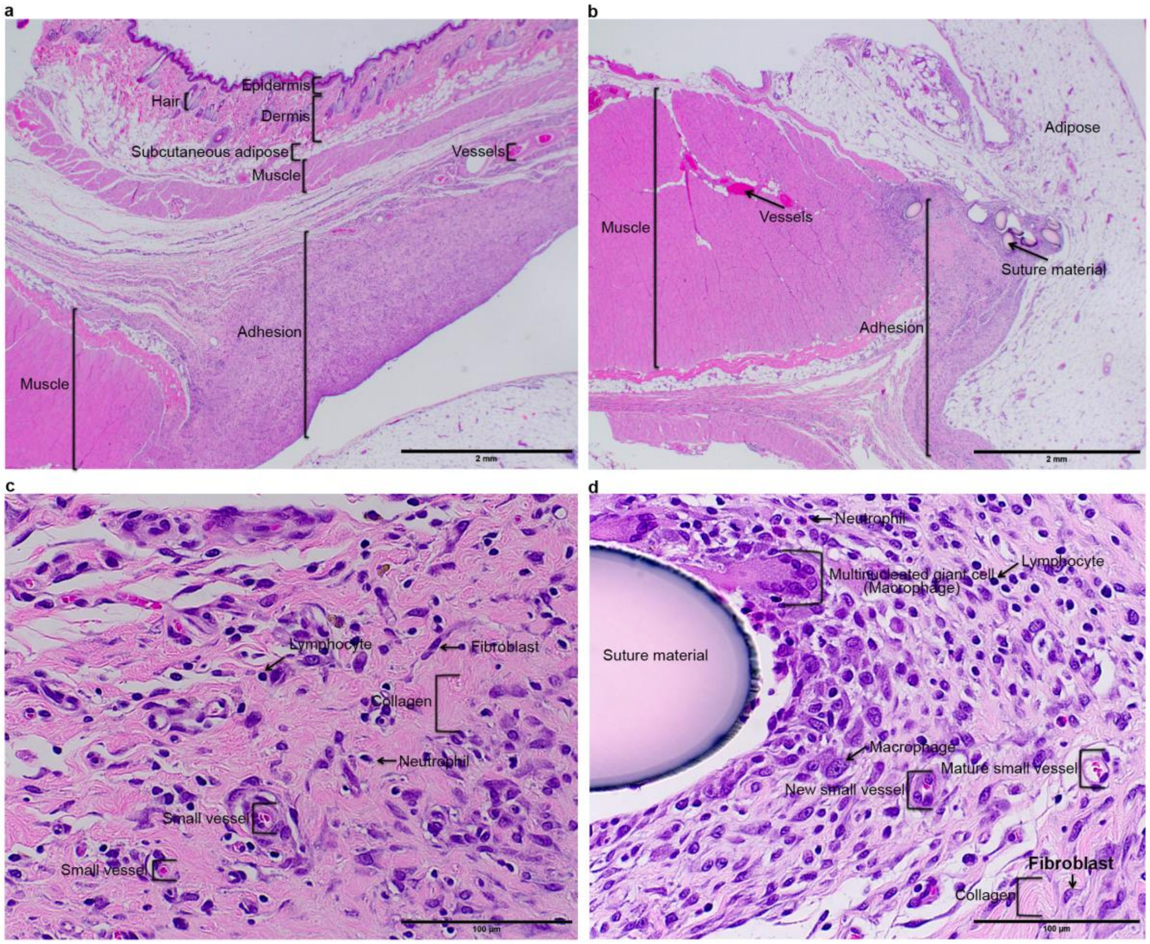

Supplementary Fig. S12. a-d, Representative H&E staining histology tissue images showing muscle and adhesion/fibrosis after abdominal surgery. For (a) and (b), scale bar = 2 mm; For (c) and (d), scale bar = 100 μm.

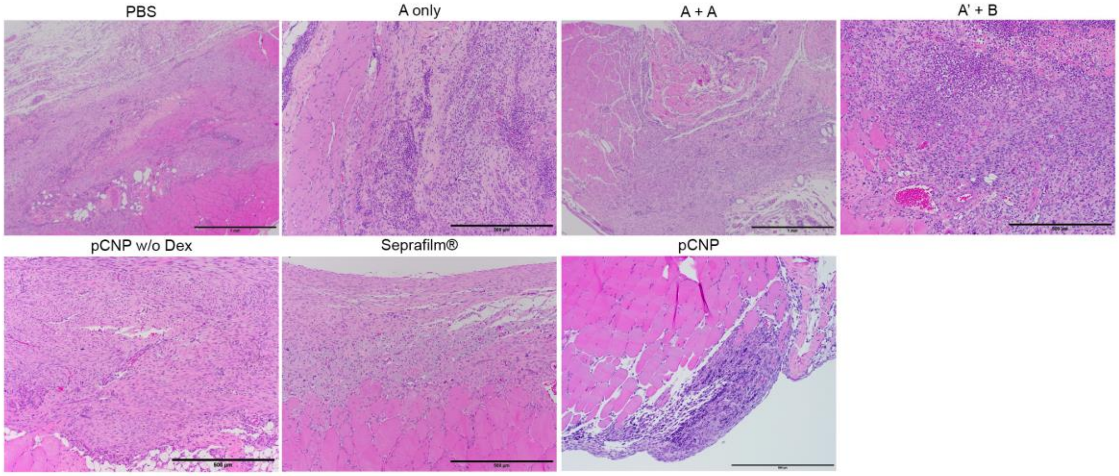

Supplementary Fig. S13. Representative H&E staining histology tissue images showing the adhesion/fibrosis after treatments. For PBS and A+A group, scale bar = 1 mm; for others, scale bar = 500 μm.

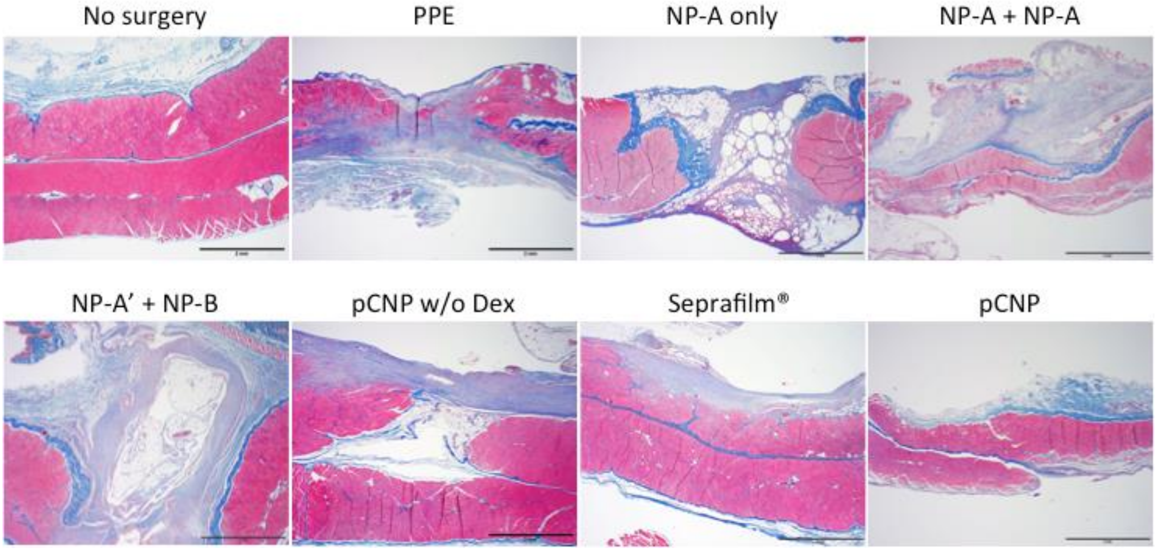

Supplementary Fig. S14. Representative Masson's trichrome staining histology tissue images in untreated rats (No surgery), and rats that underwent surgery and subsequent treatment with PBS (PPE), NP-A only, NP-A + NP-A, NP-A' + NP-B, pCNP without dexamethasone 21-palmitate (pCNP w/o Dex), Seprafilm® or pCNP. Scale bar = 2 mm.

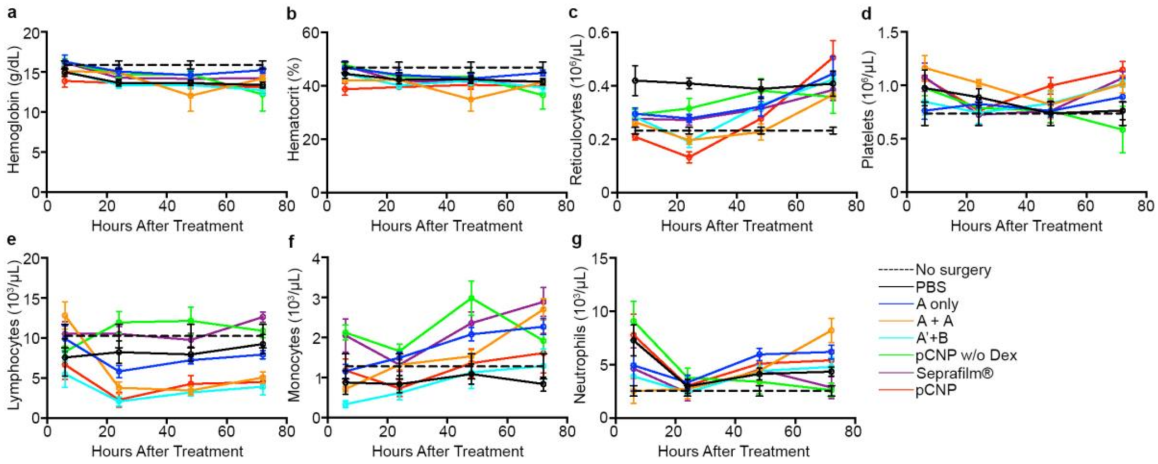

Supplementary Fig. S15. Whole blood assessment of rats at 6 h, 24 h, 48 h and 72 h after treatments. **a,** Hemoglobin count. **b,** Hematocrit count. **c,** Reticulocyte count. **d,** Platelet count. **e,** Lymphocyte count. **f,** Monocyte count. **g,** Neutrophil count. Data represents mean ± standard error of the mean (SEM). (For A+A, n=6; For pCNP w/o Dex, n=7; For Seprafilm®, n=8; For other groups, n=9).

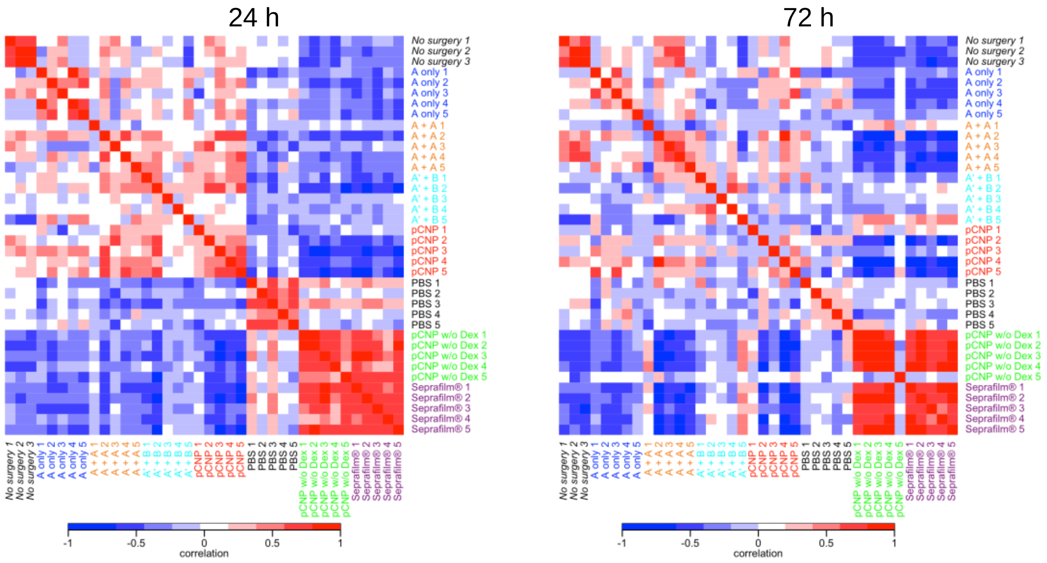

Supplementary Fig. S16. A Pearson correlation matrix of each sample versus other samples (including itself as the diagonal line) demonstrates similarities of inflammation among different treatment groups. Red indicates high positive correlation and blue indicates strong anti-correlation.

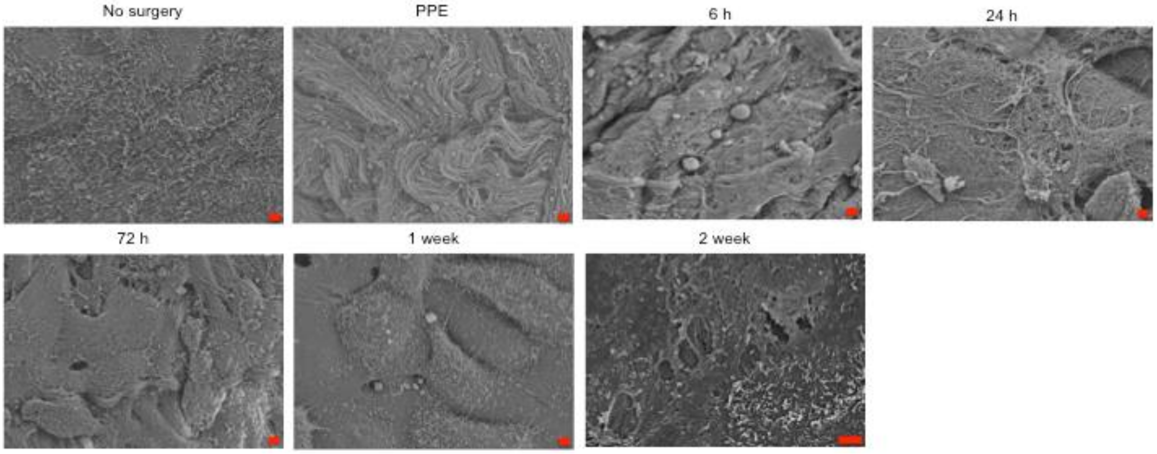

Supplementary Fig. S17. FESEM images showing the healing process of rats' abdominal wall after PPE surgery and subsequent treatment with pCNP. Scale bar = 2 μm.

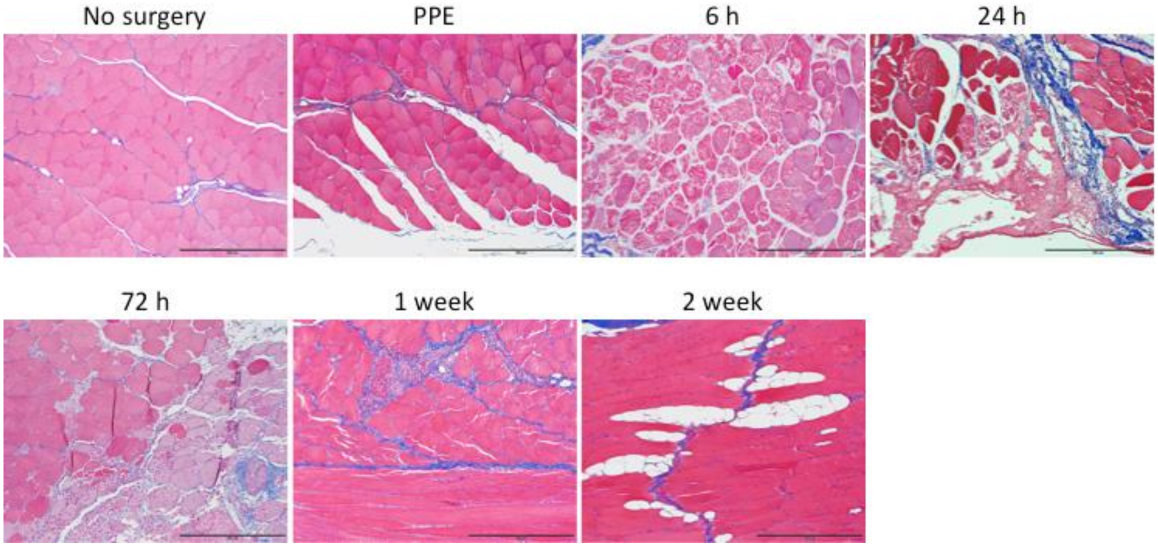

Supplementary Fig. S18. Representative Masson's trichrome staining histology tissue images on rats' abdominal wall at 6 h, 24 h, 72 h, 1 week and 2 weeks after surgery and subsequent treatment with pCNP. Scale bar = 500 µm.

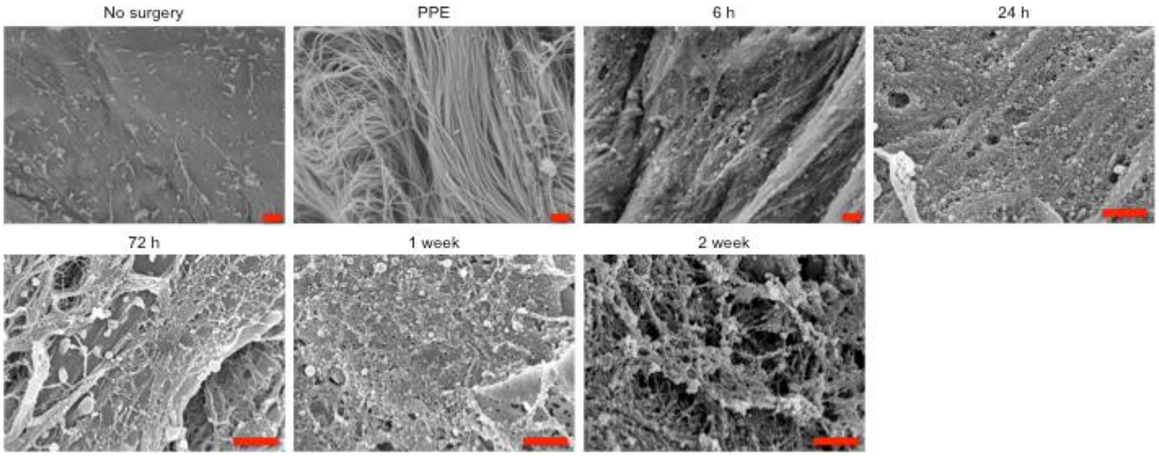

Supplementary Fig. S19. Zoom out FESEM images showing the retention and biodegradation of pCNP on rats' abdominal wall at 6 h, 24 h, 72 h, 1 week and 2 weeks after surgery and subsequent treatment with pCNP. Scale bar = 1 μm.

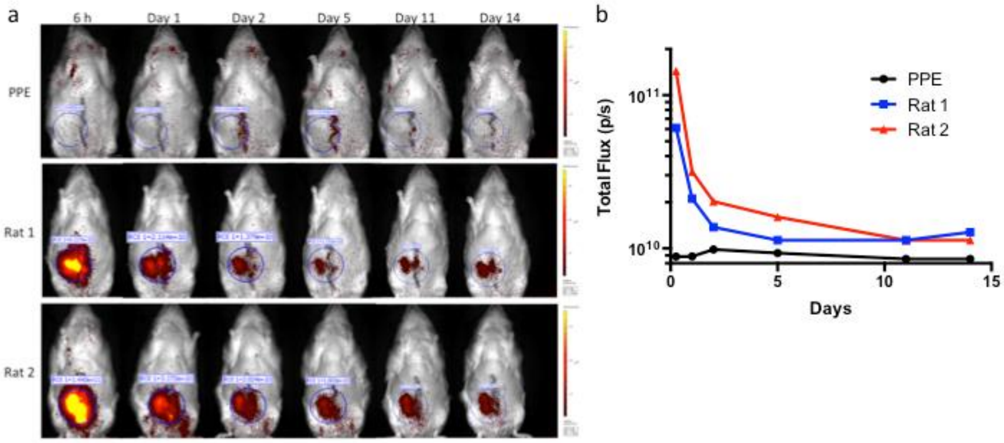

Supplementary Fig. S20. a, IVIS imaging of rats from 6 h to day 14 after surgery and treatment. Rats were excised a ~2x2 cm patch of peritoneum with the underlying muscle layer from the left abdominal wall and treated with PBS (PPE) or pCNP with NIR fluorescence (Rat1 and Rat 2). Scale bar: radiance (p/sec/cm^2/sr) from 3.5 e7 to 3.5 e8. **b,** Total flux number of circled areas in (a).

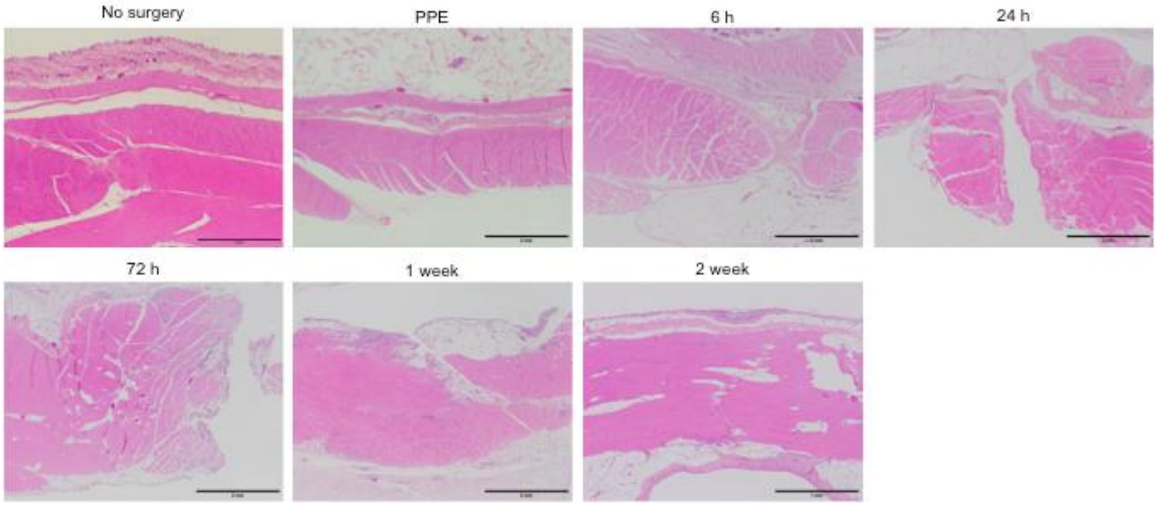

Supplementary Fig. S21. Zoom out hematoxylin and eosin (H&E) staining images of rats' abdominal wall at 6 h, 24 h, 72 h, 1 week and 2 weeks after surgery and subsequent treatment with pCNP. Scale bar = 2 mm.

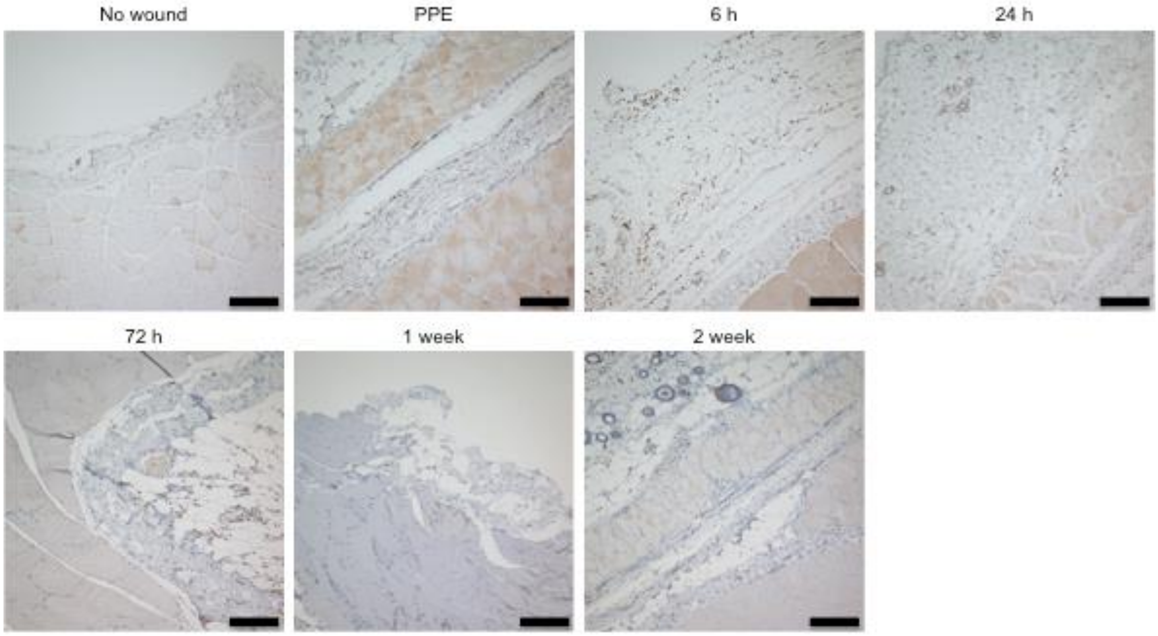

Supplementary Fig. S22. Zoom out CD45 immunohistochemistry (IHC) staining images of rats' abdominal wall at 6 h, 24 h, 72 h, 1 week and 2 weeks after surgery and subsequent treatment with pCNP. Scale bar = 200 μm.

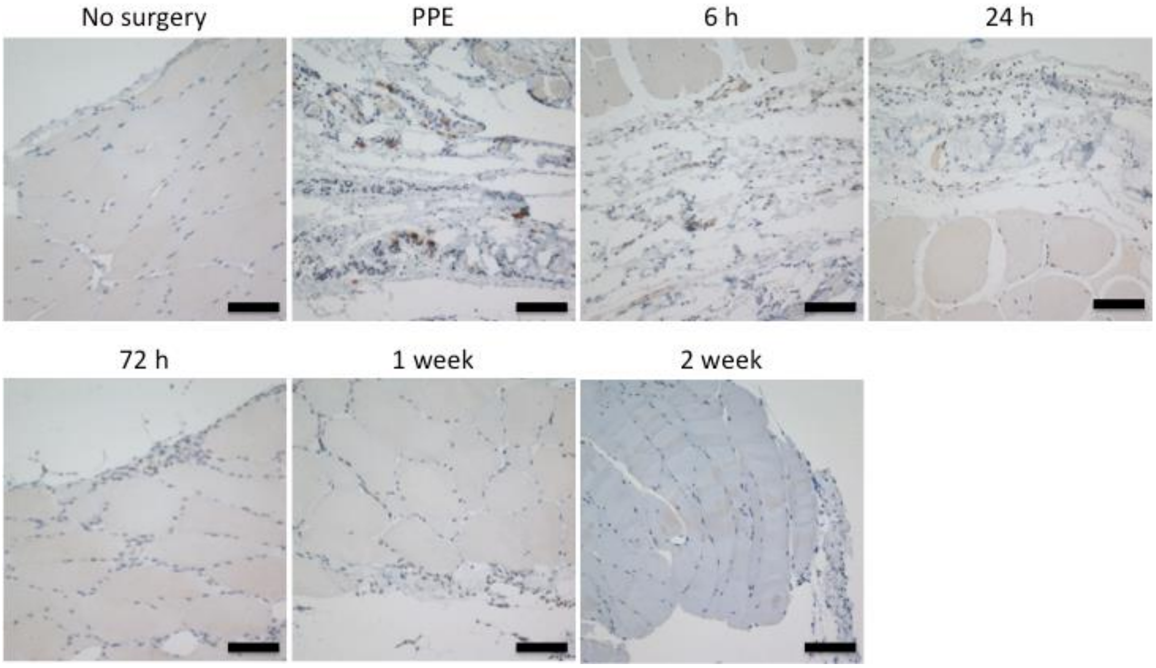

Supplementary Fig. S23. IL-1β immunohistochemistry (IHC) staining images of rats' abdominal wall at 6 h, 24 h, 72 h, 1 week and 2 weeks after surgery and subsequent treatment with pCNP. Scale bar = 100 μm.
